# The Effects of Sampling and Storage Conditions on the Metabolite Profile of the Marine Sponge *Geodia barretti*

**DOI:** 10.3389/fchem.2021.662659

**Published:** 2021-05-10

**Authors:** Ida Erngren, Eva Smit, Curt Pettersson, Paco Cárdenas, Mikael Hedeland

**Affiliations:** ^1^ Analytical Pharmaceutical Chemistry, Department of Medicinal Chemistry, Uppsala University, Uppsala, Sweden; ^2^ BioAnalytical Chemistry, Department of Chemistry and Pharmaceutical Sciences, Vrije Universiteit Amsterdam, Amsterdam, Netherlands; ^3^ Pharmacognosy, Department of Pharmaceutical Biosciences, Uppsala University, Uppsala, Sweden

**Keywords:** metabolomics, liquid chromatography–mass spectrometry, sponge (porifera), *Geodia barretti*, natural products, sampling

## Abstract

*Geodia barretti* is a deep-sea marine sponge common in the north Atlantic and waters outside of Norway and Sweden. The sampling and subsequent treatment as well as storage of sponges for metabolomics analyses can be performed in different ways, the most commonly used being freezing (directly upon collection or later) or by storage in solvent, commonly ethanol, followed by freeze-drying. In this study we therefore investigated different sampling protocols and their effects on the detected metabolite profiles in liquid chromatography-mass spectrometry (LC-MS) using an untargeted metabolomics approach. Sponges (*G. barretti*) were collected outside the Swedish west coast and pieces from three sponge specimens were either flash frozen in liquid nitrogen, frozen later after the collection cruise, stored in ethanol or stored in methanol. The storage solvents as well as the actual sponge pieces were analyzed, all samples were analyzed with hydrophilic interaction liquid chromatography as well as reversed phase liquid chromatography with high resolution mass spectrometry using full-scan in positive and negative ionization mode. The data were evaluated using multivariate data analysis. The highest metabolite intensities were found in the frozen samples (flash frozen and frozen after sampling cruise) as well as in the storage solvents (methanol and ethanol). Metabolites extracted from the sponge pieces that had been stored in solvent were found in very low intensity, since the majority of metabolites were extracted to the solvents to a high degree. The exception being larger peptides and some lipids. The lowest variation between replicates were found in the flash frozen samples. In conclusion, the preferred method for sampling of sponges for metabolomics was found to be immediate freezing in liquid nitrogen. However, freezing the sponge samples after some time proved to be a reliable method as well, albeit with higher variation between the replicates. The study highlights the importance of saving ethanol extracts after preservation of specimens for biology studies; these valuable extracts could be further used in studies of natural products, chemosystematics or metabolomics.

## Introduction

Sponges (phylum Porifera) are benthic filter-feeding animals present in most marine and fresh-water habitats (van Soest et al., 2012). They are characterized by rich and diverse prokaryotic communities, which consist of bacteria as well as archaea (Vacelet and Donadey, 1977; Preston et al., 1996). Due to the wide variety of metabolites, of which some are thought to play an important role in chemical defense Thoms and Schupp (2007), Wilson et al. (2014), Slaby et al. (2017), Tianero et al. (2019), sponges have been recognized as an interesting source of bioactive natural products (Leal et al., 2012; Carroll et al., 2019). The diversity of the prokaryotic community is thought to contribute to the wide variety of metabolites present in sponges (Ebada and Proksch, 2012; Kornprobst, 2014). *Geodia barretti* (Geodiidae family, Tetractinellida order) is a massive deep-sea demosponge that is widely spread in the North Atlantic, including the Swedish West coast (Cárdenas et al., 2013; Cárdenas and Rapp, 2015). It is a key species of boreal sponge grounds Klitgaard and Tendal (2004), Murillo et al. (2012), regularly studied for the many ecosystem services it provides Hoffmann et al. (2009), Leys et al. (2018), Maier et al. (2020) and an important research organism in sponge biology Kutti et al. (2015), Strand et al. (2017), Koutsouveli et al. (2020a), Koutsouveli et al. (2020b), de Kluijver et al. (2021), sponge cell culture Conkling et al. (2019), sponge microbiology Radax et al. (2012), Schöttner et al. (2013), Luter et al. (2017) as well as marine natural product research (Lidgren and Bohlin, 1986; Sjögren et al., 2004; Hedner et al., 2008; Carstens et al., 2015; Olsen et al., 2016a; Di et al., 2018).

In the hunt for new bioactive compounds and natural products, metabolomics, especially liquid chromatography high-resolution mass spectrometry (LC-HRMS) based analysis, has gained interest owing to its capacity to analyze large numbers of metabolites simultaneously as well as its high sensitivity, reducing the amount of biological material necessary (Paul et al., 2019). In sponge research metabolomics has been used to study differences in the metabolome depending on geographical location Bayona et al. (2020), between species Olsen et al. (2016b), Reverter et al. (2018) or to investigate the chemical diversity of sponge extracts (Cantrell et al., 2019; Mohanty et al., 2020). However, very few systematic investigations of method development for metabolomics of sponges have been performed. Bayona et al. (2018) investigated the influence of temperature and extraction solvent on the chemical diversity of the extracts by evaluating a chemical diversity index in nuclear magnetic resonance (NMR). However, very few details regarding differences in extracted metabolites or the analytical variation were investigated. Bojko et al. (2019) investigated different solid phase micro extraction (SPME) devices for the extraction of exometabolites from sponges.

Sample collection for metabolomics strive to capture the metabolic state of the sample at the time of sampling and therefore the it should alter the metabolome as little as possible as well as quench the metabolism as quickly as possible to avoid metabolite degradation post-sampling. Furthermore, factors such as sampling, quenching the metabolism, storage conditions as well as sample extraction parameters have great effects on the final metabolome as well as data quality Engskog et al. (2016), Vuckovic (2018), and have been investigated and reviewed for plasma, serum and urine Bi et al. (2020), González-Domínguez et al. (2020), Smith et al. (2020), mammalian cells Dettmer et al. (2011), Bi et al. (2013), Engskog et al. (2015), bacteria and microbes Mashego et al. (2007), Rabinowitz (2007), Kapoore and Vaidyanathan (2018), as well as for plants (Kim and Verpoorte, 2010; Rodrigues et al., 2019; Salem et al., 2020). For the collection of sponges for metabolomics, no guidelines or practical consensus exist and the procedures for sampling varies, in some cases the sponges are frozen directly upon collection, in some cases the time between collection and freezing is not stated in the publications and in some cases the sponges are immediately stored in ethanol where after the ethanol is often discarded prior to extraction of the sponge (Olsen et al., 2016b; Einarsdottir et al., 2017; Bayona et al., 2018; Bayona et al., 2020; Reverter et al., 2018; Mohanty et al., 2020).

Therefore, we aimed at investigating the influence of sampling and storage conditions on the technical variability and metabolite profiles of the marine sponge *Geodia barretti*. Specimens of *G. barretti* were collected and pieces of the sponges were either immediately frozen in liquid nitrogen, stored in ambient temperature until arrival at the marine station after the collection cruise and then frozen, stored in ethanol or stored in methanol. For the sponges stored in either ethanol or methanol, the storage solvents as well as the remaining sponge pieces were analyzed after storage. All samples were analyzed using LC-QTOF-MS with both reversed phase (RP) and hydrophilic interaction liquid chromatography (HILIC), in positive as well as negative ionization mode for a wide coverage of the metabolome.

## Material and Methods

### Chemicals

Acetonitrile (ACN, LC-MS grade), ammonium formate (LC-MS grade), formic acid (LC-MS grade), methanol (LC-MS grade) for all LC-MS analyses and metabolite extractions as well as methanol (HPLC-grade) used for sponge storage/extraction just after sampling was purchased from Fisher Scientific (Loughborough, United Kingdom). Dichloromethane (DCM, HPLC grade) was obtained from VWR (Fontenay-sous-Bois, France) and ethanol (reagent grad/HPLC grade) was purchased from Fisher Scientific (Loughborough, United Kingdom). Isopropanol, IPA (LC-MS grade) was bought from Janssen (Geel, Belgium). Ammonium hydroxide (25%, LC-MS grade) was purchased from Merck (Darmstadt, Germany).

### Sampling of *Geodia barretti*

In total three *Geodia barretti* specimens were collected in Krugglöbranten, the Koster Sea, outside Tjärnö on March 27, 2019. Specimens were identified on board by PC; collection data is presented in Table 1. Surface to bottom water temperature was 5–6°C, while air temperature was 10°C. The specimens were collected at depths between 90 and 100 m using a remotely operated vehicle (ROV) equipped with a net basket. Directly after collection, the specimens were photographed and from the center of each specimen (the cortex was avoided), 12 pieces of approximately 1 × 0.5 × 0.5 cm were sampled. Of the 12 pieces, three were directly frozen on board using liquid N_2_, three were stored in ethanol (10 ml per piece), three were stored in methanol (10 ml per piece) and three were stored at ambient temperature (45 min–2 h 5 min) until freezing in −80°C at the marine station. The solvents used for fixation of the samples were kept at ambient temperature the entire collection cruise. The flash frozen pieces in liquid N_2_ were also transferred to −80°C at the marine station. The sponge pieces stored in MeOH/EtOH were stored at approximately 20°C, until the 29th of March, when they were frozen in −20°C.

**TABLE 1:** Summary of sampling conditions for the three *Geodia barretti* specimens.

Specimen	Specimen ID	Date and time of collection	Sampling coordinates	Sampling depth (m)	Time until freezing
*G. barretti S1*	270319_02	27–03–2019	N 58°53.10495	89.3	2 h 5 m
		09:55	E 11°06.4978		
*G. barretti S2*	270319_03	27–03–2019	N 58°53.10495	90.1	1 h 50 m
		10:11	E 11°06.4978		
*G. barretti S3*	270319_05	27–03–2019	N 58°53.10495	95.1	45 m
		11:14	E 11°06.4978		

The sponge samples stored in MeOH/EtOH were transferred to fresh MeOH or EtOH respectively on the August 21, 2019 (147 days after collection), thereafter stored at −20°C until extraction. However, the solvent (extracts) in which the samples were stored in initially were saved for analysis, hereafter denoted as EtOH extr.1 and MeOH extr.1, respectively. Prior to extraction the sponges were separated from the solvents and freeze-dried, the solvents were saved for further analysis and is hereafter denoted EtOH extr.2 and MeOH extr.2 respectively. The sponges are denoted EtOH sp. and MeOH sp. respectively.

**FIGURE 1:**
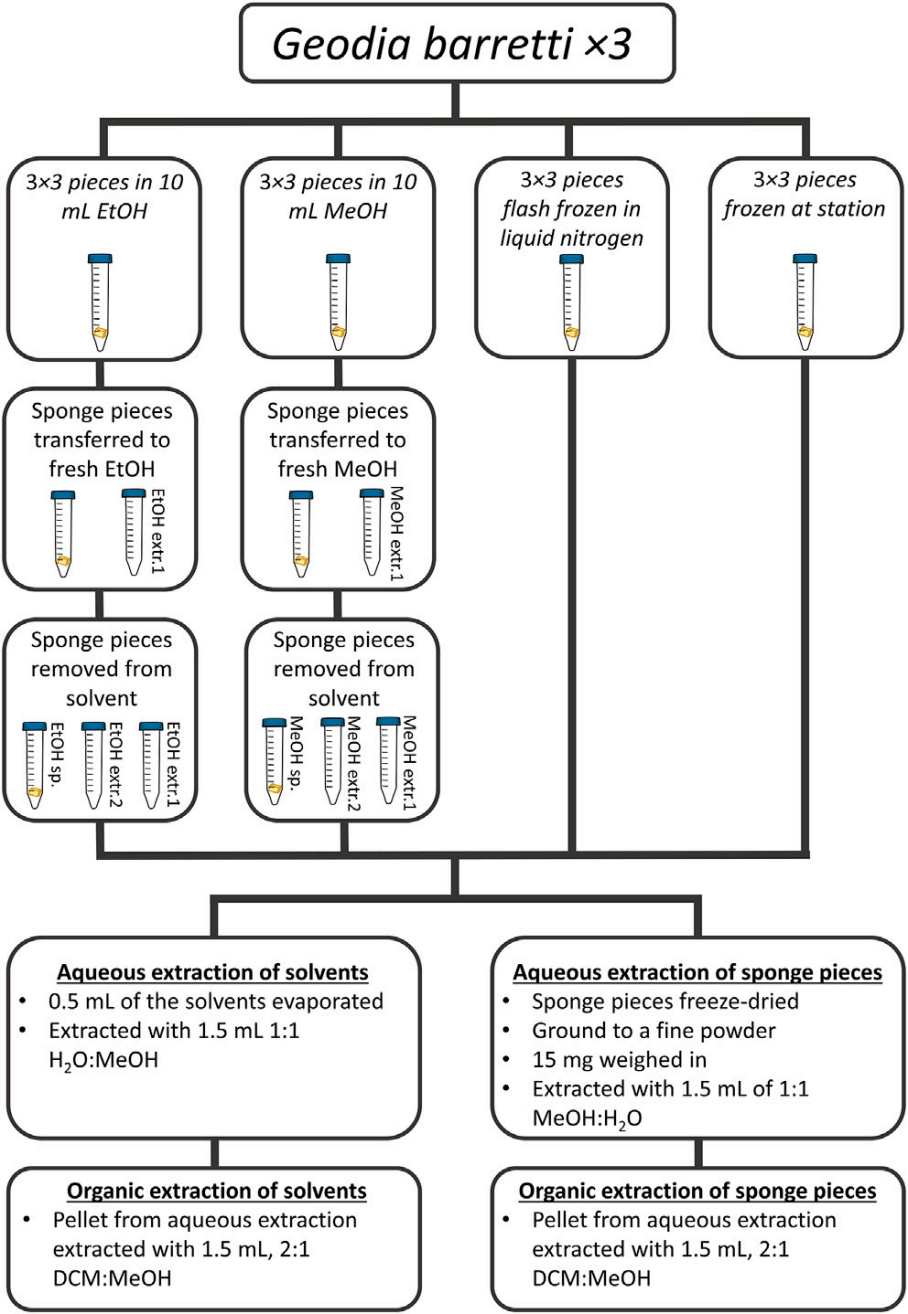
A summary of the collected samples and the treatments after sampling as well as extraction. In total three specimens of *G. barretti* were collected, from each specimen three pieces were taken for each of the treatments.

In summary, the samples were divided into eight sample types; flash frozen in liquid N_2_ (flash frozen), frozen at station (frozen), ethanol in which the sponge was stored first (EtOH extr.1), ethanol in which the sponge was stored secondly (EtOH extr.2), the sponge pieces stored in ethanol (EtOH sp.), methanol in which the sponge was stored first (MeOH extr.1), methanol which the sponge was stored in secondly (MeOH extr.2) and finally the sponge pieces stored in methanol (MeOH sp.). Each sample group included nine samples, three technical replicates from each of the three specimens (Figure 1).

### Metabolite Extraction

The sponges stored in EtOH or MeOH were separated from the liquids and all sponge samples were freeze-dried at −88°C and the pressure was kept below 500 mTorr. The freeze-dried sponge samples were stored in −80°C between freeze-drying and extraction. Just before extraction the freeze-dried samples were ground to a fine powder using tweezers and spatula. For extraction, 15 mg of freeze dried sponge was weighed in to 750 µL of 50 mM aqueous ammonium formate at pH 8 in water chilled to 8°C, the samples were vortexed and sonicated quickly, 750 µL of MeOH was added and the samples were vortexed. Ammonium formate at pH 8 was used in the aqueous extraction to ensure the pH during extraction was the same for all sample groups as the sample groups had been treated differently prior to extraction. The samples were then shaken for 30 min at 4°C and 800 RPM. After shaking, the samples were centrifuged at 4°C and 877 × g for 10 min, 1.00 ml of the aqueous supernatant was collected and evaporated in a vacuum concentrator at 30°C.

An aliquot (0.500 ml) from the liquid samples, EtOH extr.1, EtOH extr.2, MeOH extr.1 and MeOH extr.2 were evaporated prior to extraction using a vacuum concentrator (Eppendorf) at 30°C. After evaporation 750 µL of 50 mM aqueous ammonium formate at pH 8 and 750 µL MeOH was added and the samples were shaken for 30 min at 4°C and 800 RPM. After shaking, the samples were centrifuged at 4°C and 877 × g for 10 min, 1.00 ml of the aqueous supernatant was collected and evaporated in a vacuum concentrator at 30°C.

For extraction of lipophilic metabolites the remaining supernatant from the extraction of hydrophilic metabolites was evaporated until dry and 1.50 ml, chilled 2:1 DCM:MeOH was added to the dried pellet. The samples were vortexed and shaken for 30 min at 4°C and 800 RPM, then centrifuged for 10 min at 4°C and 877 × g. The organic extracts (1.00 ml) were isolated and evaporated to dryness in a vacuum concentrator at 30°C. All extracts were weighed after evaporation to assess the extraction yields and the samples were then stored at −80°C until to analysis. The aqueous extracts were reconstituted just prior to HILIC-ESI-HRMS analysis in 75 µL water and 1,425 µL ACN and vortexed before they were transferred to LC-vials. The organic extracts (lipophilic metabolites) were reconstituted just prior to RPLC-ESI-HRMS analysis in 100 µL of 95:5 water:ACN and vortexed before they were transferred to LC-vials. For the aqueous extracts and organic extracts respectively a QC sample was created by pooling aliquots from each sample.

### Liquid Chromatography-Mass Spectrometry Analysis

All samples were analyzed on an Acquity UPLC (I-class, Waters, Milford, MA, United States) hyphenated to a Synapt G2-S Q-TOF mass spectrometer (Waters) in both positive and negative ionization mode using electrospray ionization, all systems were controlled by Masslynx 4.1 (Waters). The hydrophilic metabolites were analyzed in HILIC-ESI-MS on a Acquity BEH Amide column (1.7 µm, 2.1 × 50 mm, Waters). For the HILIC analysis, mobile phase A comprised of 5 mM ammonium formate with 0.025% formic acid in 95:5 ACN:water and mobile phase B comprised of 5 mM ammonium formate with formic acid in 40:60 ACN:water. The elution gradient was non-linear from 100% A to 100% B over 14 min, followed by isocratic separation at 100% B for 2 min, then a linear decrease back to 100% A over 1 min and re-equilibration at 100% A for 6 min. The non-linear gradient (slope factor 8 in Masslynx) was defined as follows C(t) = Ci+(Cf−Ci)*X^3^, where X=(t−Ti)/(Tf−Ti) and C(t) are the composition C in percent at time t, Ci the initial mobile phase composition, Cf the final mobile phase composition, Ti and Tf the initial and final times respectively. The mobile phase flow rate was set to 0.4 ml min^−1^, the column temperature was set to 40°C and the injection volume was 5 µL.

The organic extracts (lipophilic metabolites) were analyzed in RP-LC-MS using an Acquity BEH C18 column (1.7 µm, 2.1 × 50 mm, Waters). In the RP analysis, mobile phase A was 85:15 water:ACN with 0.1% formic acid and mobile phase B was 30:70 ACN:IPA. The elution gradient was non-linear from 100% A to 98% B over 15 min, followed by 4 min at 98% B and then decreased to 100% A over 0.1 min followed by 3 min at 100% for re-equilibration. The non-linear gradient (slope factor 7 in Masslynx) was defined as follows C(t) = Ci+(Cf−Ci)*X^2^. The mobile phase flow rate was set to 0.3 ml min^−1^, the column temperature was 40°C and the injection volume 5 µL.

In both HILIC and RPLC the mass spectrometer was operated in resolution mode in the range *m/z* 50–1500 using full scan. The data was collected in centroid mode, with a scan time of 0.3 s and a collision energy of 4 eV. In positive/negative ionization mode the capillary voltage was set to 1 kV/1.5 kV, the cone voltage to 30 V/25 V and the source offset was set to 50 V/60 V. In both positive and negative ionization the source temperature was set to 120°C and the desolvation temperature was 500°C. Nitrogen was used as both desolvation gas and cone gas at 800 and 50 L h^−1^ respectively. Lock-spray correction (real time mass correction) was applied in all analyzes by infusing 10 μL/min leucine-enkephalin (*m/z* 556.2771 in positive mode, *m/z* 554.2615 in negative mode). MS/MS experiments were performed using the same settings as described above if not stated otherwise, however, using MS/MS mode instead of MS full-scan. The scan time of 0.5 s was set to, the collision energy at 10 eV and the quadrupole mass window set to 1 m*/z*. For identification, retention time as well as MS/MS spectra were matched.

Prior to analysis of all samples in HILIC and RPLC in both positive and negative ionization mode the respective QC sample was injected 10 times to ensure stable chromatographic and mass spectrometric conditions before analyzing the study samples. The study samples were then analyzed in randomized order with the QC samples interspaced every 6th injection to be able to monitor the analytical performance throughout the entire analysis with regard to mass accuracy, retention time drift as well as sensitivity [37]. Solvent blanks and extraction blanks were analyzed before and after each run to assess the background of the instrument as well as possible extraction contaminants.

### Data Analysis

All data files were converted to NetCDF files using Databridge (part of Masslynx software), then processed with the R-based program XCMS (version 3.8.2) (Smith et al., 2006; Tautenhahn et al., 2008; Benton et al., 2010). The QC samples were used to adjust the processing parameters for each individual dataset, HILIC positive, HILIC negative, RP positive and RP negative. The parameters were adjusted manually using the QC samples to minimize the variation within the datasets (QC samples), to control the number of missing peaks and the deviation in retention time between samples. For all datasets the centwave function was used for peak-picking, the obiwarp function for peak alignment, the peak density function was used for peak grouping and fillpeaks was used to avoid missing values in the datasets. The final datasets contained information on all the detected peaks (*m/z*-retention time) and their respective peak area in all samples. Detailed information on all used parameters can be found in the supplementary data, Supplementary Table S1 *.* After pre-processing the datasets were exported to Microsoft Excel and the datasets were filtered so that all features (*m/z*-retention time pairs) with a retention below 45 s was removed, all features with missing values in any of the QC samples were removed and all features with a coefficient of variation (CV %) above 30% was removed. After feature filtering the datasets were exported to SIMCA 16 (Umetrics, Umeå, Sweden) and the data was pareto scaled, for principal component analysis (PCA) the data was also log-transformed. In general PCA, scores plots as well as loadings plots were used to study sample clustering, trends in injection order, biological as well as technical variation and identifying eventual outliers. Analysis of similarities (ANOSIM) was used to study the between group/cluster and within group/cluster variation in the PCA models. For the ANOSIM analysis the vegan package in R was used and the analysis was performed with 999 permutations and Euclidean distances. Then orthogonal projection to latent structures-discriminant analysis (OPLS-DA) was used to find discriminating features between sample groups. During the analysis with OPLS-DA, three different models were created for each analysis mode; i) comparing all MeOH groups (MeOH extr.1, MeOH extr.2 and MeOH sp.), ii) comparing all EtOH groups (EtOH extr.1, EtOH extr.2 and EtOH sp.) and finally iii) comparing EtOH extr.1 and MeOH extr.1 to the frozen and flash frozen. The OPLS-DA models were validated using analysis of variance (ANOVA) of the cross-validated residuals (CV-ANOVA) as well as permutation testing (*n* = 100) of the Y-variable and assessing the Q^2^ and R^2^Y intercepts. In each model the Variable Importance for the Projection (VIP)-plot was used to select the 100 features with the highest VIP-scores. The selected features were annotated based on accurate mass, when possible fragmentation (in-source fragmentation), isotopic patterns as well as retention time. In short, after selection the features were examined in the raw data to study isotopic patterns and possible adduct formation as well as in-source fragmentation, thereafter the accurate mass was used for database searches to find plausible candidates that were matched with the isotopic patterns, fragmentation and adduct formation. A maximum mass error of 10 ppm was allowed for annotation. The databases used for annotation was Dictionary of Marine Natural Products, Metlin Tautenhahn et al. (2012), HMDB Wishart et al. (2007), Wishart et al. (2013), Wishart et al. (2018), and ECMDB (Sajed et al., 2016). When possible, the metabolites were identified using MS/MS of standards (see *Liquid Chromatography-Mass Spectrometry Analysis*). Differences in peak area between the different sample groups were tested for significance using a Kruskal-Wallis test and a Nemenyi post-hoc test for multiple comparisons with the PMCMRplus package in R.

## Results and Discussion

As researchers in the field of natural products research realized the potential of metabolomics as a tool for the screening of interesting secondary metabolites, the need for standardized methods for sample collection, treatment, storage, preparation and analytical validation has arisen. In the current study we aimed at investigating the impact of different collection and storage methods of the sponge *Geodia barretti* on the metabolome as well as analytical outcomes such as data quality and possibilities for relative quantifications. In total three specimens of the sponge *G. barretti* were collected in the Koster Sea outside the west coast of Sweden and from each specimen three technical replicates were stored in ethanol, methanol, frozen directly with liquid nitrogen or frozen back at the station after the collection cruise (after 45 min to 2 h), respectively, (for details see Table 1; Figure 1). The solvents in which the sponges were stored in were exchanged twice but saved for further analysis resulting in eight different sample groups.

### Extraction of Hydrophilic and Lipophilic Metabolites

**FIGURE 2:**
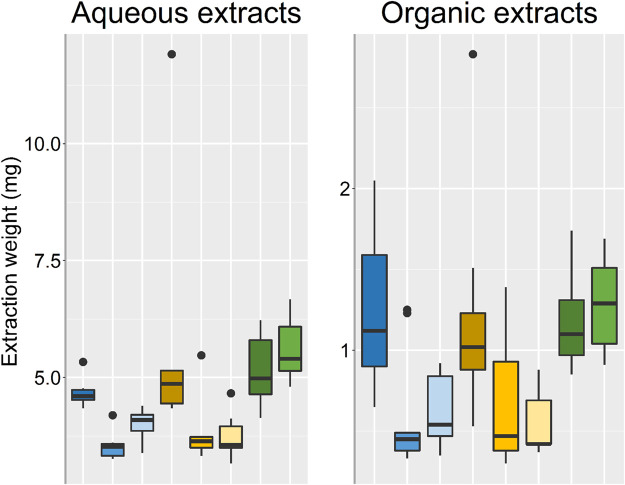
Box plots over the respective extraction weights for each sample group, outliers are denoted by dots. EtOH extr.1 (dark blue 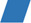), EtOH extr.2 (blue 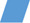), EtOH sp. (light blue 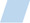), MeOH extr.1 (dark orange 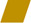), MeOH extr.2 (yellow 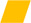), MeOH sp. (light yellow 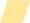), frozen (dark green 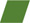), and flash frozen (green 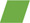).

A difficult aspect of comparing the different groups in terms relative abundances of metabolites and extraction of metabolites, was how extraction parameters and ratios should be scaled for a fair comparison between groups. The EtOH/MeOH extr.1 and frozen/flash frozen samples gave rise to the highest relative abundances of metabolites during initial analyses (for comparison see supplementary data Supplementary Figures S1–S4). Therefore, the volumes of the EtOH/MeOH extr.1 and the weighed in amounts of sponge from the frozen/flash frozen samples were adapted so that the weights of the dried extracts (after aqueous extraction), were of the same order of magnitude (Figure 2). In Figure 2, the weights of the extracts, both aqueous and organic, are presented, each group consisting of nine samples in total, three technical replicates from three specimens. The extraction weights of the EtOH extr.1, MeOH extr.1 and frozen samples groups were similar, ranging from 4.60 to 4.98 mg (median), while the extraction weights of the flash frozen sample group was slightly higher (median 5.40 mg), however, not significantly. The sponge samples that had been stored in EtOH or MeOH both had lower extraction weights than the frozen/flash frozen sponges, even though the same amount of freeze-dried sponge material was weighed in (15 mg) before the extraction, suggesting a substantial extraction from the sponge pieces to solvents they had been stored in. In both the aqueous extracts and the organic extracts one sample in the MeOH extr.1 group deviated, which was found later to be due to insufficient evaporation after extraction. However, the sample did not deviate in the data analysis or multivariate data analysis and was therefore not excluded. The extraction weights of the organic extracts were lower than those of the aqueous extracts, which could be expected as a large amount of the metabolites had already been extracted to the aqueous phase and no buffer salts that can add to the extraction weights, were used in the organic extraction solvent. However, the relative weights between the sample groups were very similar in the two extractions (Figure 2).

### Multivariate Data Analysis

Each analysis mode was processed separately, resulting in four datasets, i.e., two datasets for the aqueous extracts analyzed with HILIC-QTOF-MS in positive and negative ionization mode respectively and two datasets for the organic extracts analyzed with RPLC-QTOF-MS in positive and negative ionization mode respectively. To assess the analytical stability throughout the analysis the coefficients of variance (CV) of peak areas were calculated for each feature in the QC samples, interspaced between the study samples and injected every 6th injection, and all features with a CV above 30% were excluded from the datasets. In the aq. negative, aq. positive, org. negative and org. positive datasets, 96, 98, 93, and 87% of the features respectively had a CV (%) of peak area in the QC samples below 30% (Supplementary data, Supplementary Table S2). The datasets were further filtered to only contain features that were found in all QC samples. The final datasets included 971, 2,138, 1,560, and 2,522 features in aq. negative, aq. positive, org. negative and org. positive, respectively, (Supplementary data, Supplementary Table S2). Furthermore, the retention time drift for three metabolites in each analysis mode was studied in the raw data, the maximum observed retention time drift was 0.01 min in the HILIC analyses and 0.03 min in the RP analyses*.* Consequently, the analytical conditions were stable throughout all analyses and data analysis was continued.

#### Principal Component Analysis

**FIGURE 3:**
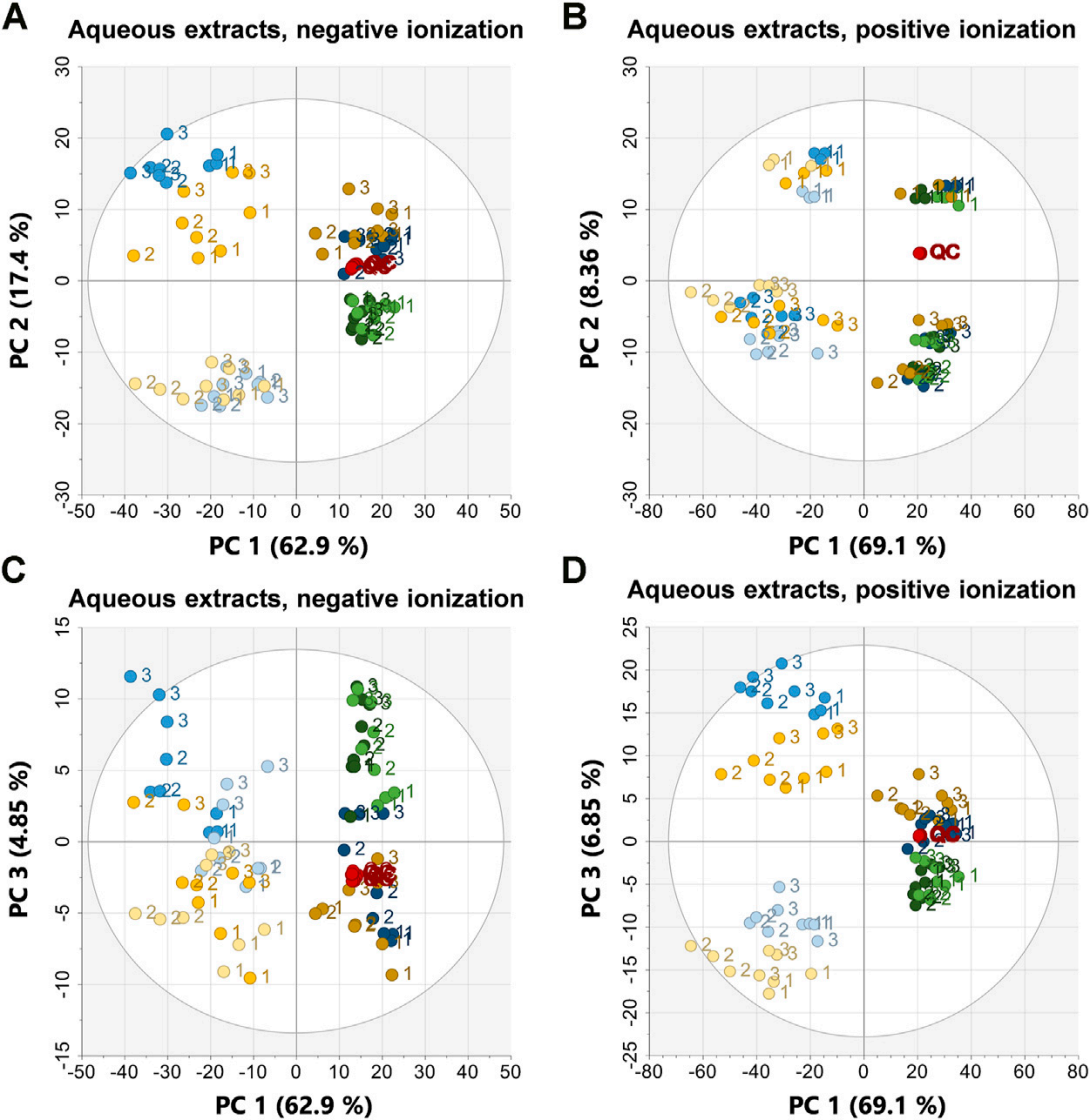
PCA scores plots of the aqueous extracts in negative ionization (R2X = 0.974, Q2 = 0.949) to the left and positive ionization (R2X = 0.966, Q2 = 0.938) on the right. **(A)** PC1 vs. PC2 in negative ionization, **(B)** PC1 vs. PC2 in positive ionization, **(C)** PC1 vs. PC3 in negative ionization, **(D)** PC1 vs. PC3 in positive ionization. Labels denote the specimen id:s 1, 2, and 3. EtOH extr.1 (dark blue 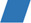), EtOH extr.2 (blue 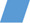), EtOH sp. (light blue 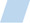), MeOH extr.1 (dark orange 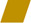), MeOH extr.2 (yellow 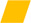), MeOH sp. (light yellow 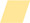), frozen (dark green 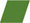) and flash frozen (green 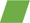), and QC-samples (red 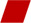).

**FIGURE 4:**
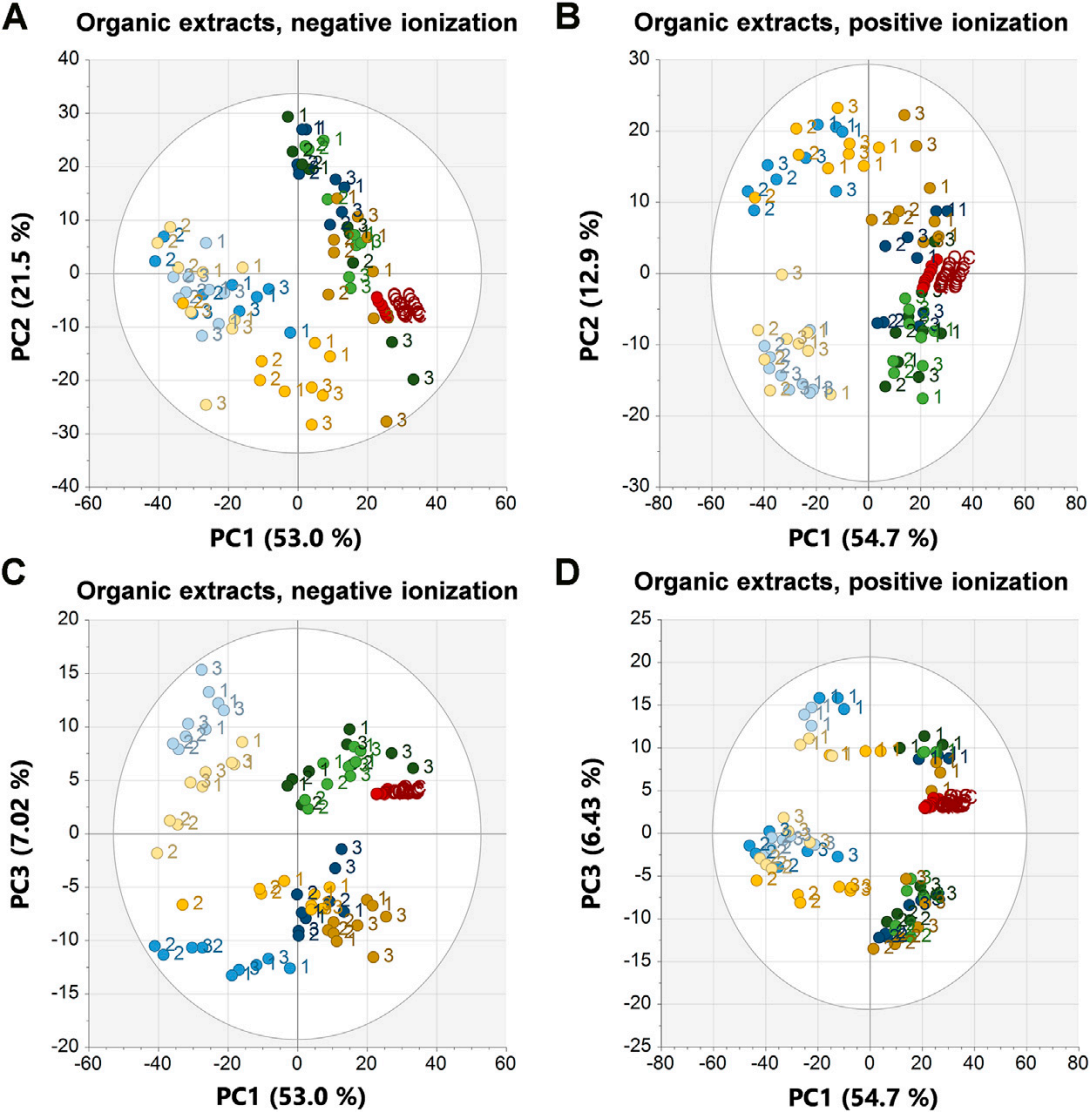
PCA scores plots of the organic extracts in negative ionization (R2X = 0.958, Q2 = 0.912) to the left and positive ionization (R2X = 0.937, Q2 = 0.887) to the right. **(A)** PC1 vs. PC2 in negative ionization, **(B)** PC1 vs. PC2 in positive ionization, **(C)** PC1 vs. PC3 in negative ionization, **(D)** PC1 vs. PC3 in positive ionization. Labels denote the specimen id:s 1, 2, and 3. EtOH extr.1 (dark blue 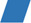), EtOH extr.2 (blue 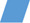), EtOH sp. (light blue 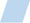), MeOH extr.1 (dark orange 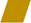), MeOH extr.2 (yellow 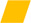), MeOH sp. (light yellow 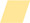), frozen (dark green 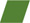) and flash frozen (green 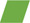), and QC-samples (red 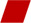).

To investigate sample clustering and grouping PCA scores plots were used for all datasets (Figures 3, 4), the corresponding loadings plots are presented in the supplementary data Supplementary Figures S5, S6. In all PCA models the QC samples clustered very closely together, in the organic positive PCA scores plot, the QC samples drift slightly due to injection order and that was also the analysis mode with the highest variation within the dataset and the largest retention time drift (Figure 4B). To test the clustering of samples in the PCA scores plots statistically, analysis of similarities, ANOSIM was used, the clustering of sample groups as well as the clustering of sample types, i.e., frozen samples, first extracts etc. were investigated (Table 2).

**TABLE 2:** Summary of the results from ANOSIM testing to evaluates the clustering in PCA scores plots. The significance levels for the ANOSIM was set to *p* = 0.001 and the analysis was performed comparing the clustering of sample groups as well as sample types, i.e., frozen samples, first extracts etc.

PCA model		R sample group	R sample type
Aqueous extracts, negative ionization	PC1 vs. PC2	0.8121	0.9108
	PC1 vs. PC3	0.7073	0.7799
Aqueous extracts, positive ionization	PC1 vs. PC2	0.5588	0.6516
	PC1 vs. PC3	0.7267	0.8152
Organic extracts, negative ionization	PC1 vs. PC2	0.5951	0.5863
	PC1 vs. PC3	0.6953	0.7044
Organic extracts, positive ionization	PC1 vs. PC2	0.7328	0.8409
	PC1 vs. PC3	0.5320	0.6300

In all PCA models, along principal component (PC) 1 the first extracts of EtOH/MeOH and the frozen sponge pieces (frozen + flash frozen) clustered together and were separated from the second extracts EtOH/MeOH and the sponge pieces stored in EtOH/MeOH. The first extracts of EtOH/MeOH and frozen as well as flash frozen sample groups were the most metabolite abundant sample groups. This was reflected in higher extraction weights but also higher intensities in the raw data and base peak intensity (BPI) chromatograms (Figure 2; supplementary data Supplementary Figures S1–S4). Along PC2 in the aqueous extracts in negative ionization the extraction liquids (EtOH/MeOH extr.1 and extr.2) were separated from the sponge samples (frozen, flash frozen, EtOH sp. and MeOH sp.) (Figure 3A). This suggests that there are differences in the metabolites extracted from the freeze-dried sponges and the fresh sponges, e.g. some metabolites might not be extracted unless the sponge sample is freeze-dried and mechanically processed. In the aqueous positive PCA scores plot, specimen 1 was separated from the two other specimens along PC2, i.e. biological variation was behind the separation along PC2 (Figure 3B). Investigating the loadings plot (Supplementary data Supplementary Figure S5) it was observed that it was mainly a few metabolites, much higher in intensity in specimen 1 that caused the separation: those were 6-bromoconicamin, 8-hydroxy-6-bromoconicamin, L-6-bromohypaphorine and an unknown brominated metabolite X457 (*m/z* 458.129 [M + H]^+^). These observations were also confirmed by the ANOSIM (Table 2) where a high correlation for the clustering of both the sample groups and sample types were very strong in the aqueous extracts, negative ionization score plots but weaker in the score plots for positive ionization, especially the PC1 vs. PC2 scores plot in positive ionization.

The metabolites mentioned as the main reason for the separation along PC2 in aqueous positive, e.g., 6-bromoconicamin, 8-hydroxy-6-bromoconicamin, L-6-bromohypaphorine are all quaternary ammonium compounds and very high in intensity in the positive ionization. However, they do not ionize very well in negative ionization which could be the reason as to why the same separation was not observed in negative ionization (Figure 3A). The reason behind the difference between specimen 1 and the other specimens has not been investigated in depth. However, all specimens were of approximately the same size, the pieces were sampled from the interior of the sponges and all specimens were collected during the same day from the same region (geographically) and depth. Furthermore, the reproductive status of the specimens were controlled by using histological sections but none of the three samples were in a reproductive state (Koutsouveli et al., 2020a). No separation between the EtOH extr.1 and MeOH extr.1 was detected in the PCA:s of the aqueous extracts (Figure 3). However, in the PCA score plots of the aqueous extracts, some separation between EtOH extr.2 and MeOH extr.2 could be observed as well as separation between EtOH sp. and MeOH sp.

The samples clustered and separated similarly in the organic extracts with some exceptions, separation along PC1 was, as in the aqueous extracts, between first extracts of EtOH/MeOH and the frozen sponge pieces (frozen + flash frozen) from the second extracts EtOH/MeOH and the sponge pieces stored in EtOH/MeOH (Figure 4). However, in negative ionization, PC2 separated mainly on biological variation and in this case it was the specimen 3 that was slightly different from the rest (Figure 4A). The separation was not caused by a few metabolites, but rather a large number of late eluting lipid species with slightly higher intensity in the deviating specimen 3. Specimen 1 was deviating along PC3 in the organic positive scores plot and the separation was once again due to a few metabolites; 6-bromoconicamin, 8-hydroxy-6-bromoconicamin, L-6-bromohypaphorine and an unknown brominated metabolite X457 (*m/z* 458.129 [M + H]^+^) (Figure 4D). The clustering of sample groups and sample types were generally less evident in the organic extracts than in the aqueous extracts which was reflected in the ANOSIM testing as well (Table 2).

**FIGURE 5:**
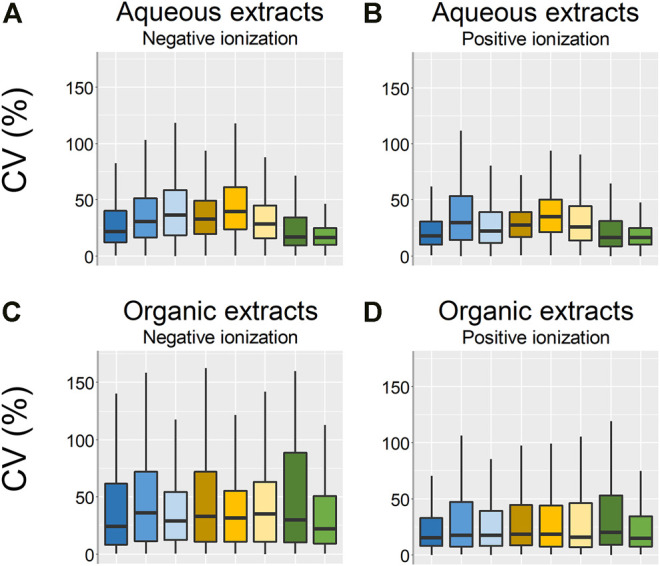
Boxplots over the CV:s (%) of the technical replicates in each sample group. **(A)** Aqueous extracts, negative ionization, **(B)** aqueous extracts positive ionization, **(C)** organic extracts negative ionization, **(D)** organic extracts positive ionization. EtOH extr.1 (dark blue 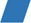), EtOH extr.2 (blue 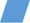), EtOH sp. (light blue 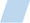), MeOH extr.1 (dark orange 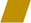), MeOH extr.2 (yellow 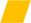), MeOH sp. (light yellow 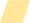), frozen (dark green 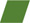), and flash frozen (green 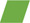).

In the PCA scores plots, there were small differences between the sample groups in terms of how tightly clustered the different samples groups were, where the frozen and flash frozen samples were slightly more tightly clustered than the rest of the sample groups (Figures 3, 4). However, no conclusions on the variation within each sample group could be drawn based only on the PCA scores plots. Therefore, the variation in peak area for each feature within the respective sample groups was calculated as CV (%) (Figure 5). The lowest variation, median CV % and interquartile range was found in the flash frozen group and in the aqueous extracts, the frozen sample group had a very similar median as the flash frozen, however, a larger interquartile range. In the organic extracts, it was the EtOH extr.1 sample group that was similar to the flash frozen regarding the median CV (%) in the entire dataset, however, again with a larger interquartile range. The lower variation of the flash frozen sample group as compared to the rest was expected, as the freezing in liquid N_2_ means that the samples were frozen from the time of sampling until extraction and that the metabolism was quenched directly after sampling in contrast to the frozen sample group. However, the small differences between the frozen and flash frozen group was somewhat surprising as the frozen group was not immediately frozen but was stored in ambient temperature (∼10°C) in quite different periods of time (45 min–2 h 5 min). That suggests that the immediate freezing of the flash frozen samples might not have such a large impact as initially thought and that the metabolism of the sponges are relatively slow. The variation in the organic datasets were larger than in the aqueous datasets, however, the differences in variation, CV (%), between the sample groups was smaller in the organic extracts.

In conclusion, judging from the PCA score plots the major differences in the metabolite profiles of the different sample groups seem to be between i) the frozen, flash frozen, EtOH extr.1, MeOH extr.1 and ii) the EtOH extr.2, MeOH extr.2, EtOH sp., MeOH sp., i.e., between the groups with the highest and the lowest signal intensity for a majority of the metabolites (discussed in more detail in *Differences in the Metabolite Profiles*) as well as higher intensity in the BPI chromatograms (supplementary data, Supplementary Figures S1–S4)*.* Furthermore along PC2, or PC3 depending on model, the sponges were separated from the extraction solvents, indicating that there were metabolites not extracted to the EtOH/MeOH but that rather required freeze-drying and/or mechanical processing for extraction.

#### OPLS-DA Analysis

In the PCA analysis differences in metabolite signal intensity between the sampling techniques was evident and OPLS-DA was used to further explore the differences between the sample groups. To investigate what was extracted into the first and second extracts of EtOH as well as MeOH but also what was left in the sponge pieces stored in the respective solvents, one OPLS-DA model for each of the respective solvents was created. One OPLS-DA model to investigate the differences between the frozen sponge samples (frozen and flash frozen) and the EtOH/MeOH first extracts was also created to investigate what was extracted by the solvents. The same three OPLS-DA models were created for all four datasets and a summary of the model parameters are presented in Table 3. The CV-ANOVA and permutation tests indicated that all models were reliable, not prone to overfitting and with good predictive power.

**TABLE 3:** Summary of quality parameters from the OPLS-DA models.

Analysis mode	Compared groups	No. of components	R2X/R2Y	Q2	*p*-value from cross validation ANOVA	Q^2^ and R^2^Y intercepts from permutation testing
Aqueous extracts, negative ionization	EtOH extr.1 vs. EtOH extr.2 vs. EtOH sp	2 + 1 + 0	0.920/0.874	0.834	2.14 × 10^−12^	Q^2^ intercept: −0.402
						R^2^ *Y* intercept: 0.106
	MeOH extr.1 vs. MeOH extr.2 vs. MeOH sp	2 + 8 + 0	0.986/0.979	0.862	3.60 × 10^−4^	Q^2^ intercept: −1.01
						R^2^ *Y* intercept: 0.8
	EtOH extr.1 and MeOH extr.1 vs. frozen and LiqN_2_	1 + 3 + 0	0874/0.909	0.866	1.76 × 10^−9^	Q^2^ intercept: −0.679
						R^2^ *Y* intercept: 0.361
Aqueous extracts, positive ionization	EtOH extr.1 vs. EtOH extr.2 vs. EtOH sp	2 + 1 + 0	0.907/0.942	0.916	1.27 × 10^−18^	Q^2^ intercept: −0.381
						R^2^ *Y* intercept: 0.103
	MeOH extr.1 vs. MeOH extr.2 vs. MeOH sp	2 + 1 + 0	0.976/0.817	0.766	2.00 × 10^−8^	Q^2^ intercept: 0.413
						R^2^ *Y* intercept: 0.145
	EtOH extr.1 and MeOH extr.1 vs. frozen and LiqN2	1 + 4 + 0	0.844/0.944	0.848	7.04 × 10^−8^	Q^2^ intercept: 0.785
						R^2^ *Y* intercept: 0.627
Organic extracts, negative ionization	EtOH extr.1 vs. EtOH extr.2 vs. EtOH sp	2 + 3 + 0	0.957/0.863	0.766	3.65 × 10^−6^	Q^2^ intercept: −0.677
						R^2^ *Y* intercept: 0.315
	MeOH extr.1 vs. MeOH extr.2 vs. MeOH sp	2 + 1 + 0	0.840/0.741	0.636	1.09 × 10^−5^	Q^2^ intercept: −0.438
						R^2^ *Y* intercept: 0.154
	EtOH extr.1 and MeOH extr.1 vs. frozen and LiqN2	1 + 3 + 0	0.750/0.937	0.885	6.44 × 10^−10^	Q^2^ intercept: −0.709
						R^2^ *Y* intercept: 0.354
Organic extracts, positive ionization	EtOH extr.1 vs. EtOH extr.2 vs. EtOH sp	2 + 1 + 0	0.871/0.790	0.712	3.11 × 10^−8^	Q^2^ intercept: −0.474
						R^2^ *Y* intercept: 0.178
	MeOH extr.1 vs. MeOH extr.2 vs. MeOH sp	2 + 1 + 0	0.789/0.716	0.507	0.003	Q^2^ intercept: −0.411
						R^2^ *Y* intercept: 0.207
	EtOH extr.1 and MeOH extr.1 vs. frozen and LiqN2	1 + 6 + 0	0.894/0.984	0.873	5.06 × 10^−7^	Q^2^ intercept: −0.799
						R^2^ *Y* intercept: 0.812

From each model the top 100 features from the VIP plot were selected and annotated. The VIP plot summarizes the importance of each variable for the projection and high VIP-scores indicate that the variables are important for the projection, i.e., the discrimination between groups. In general annotation of metabolites in sponge samples is very challenging as little is known about the sponge metabolome and many metabolites are species specific. Therefore, the previously known and reported metabolites of *G. barretti* were annotated in the datasets as well, regardless if they were within the top 100 in the VIP plots; in most cases they were represented in the initial selection, however, not in all models (Lidgren and Bohlin, 1986; Carstens et al., 2015; Olsen et al., 2016a; Di et al., 2018). All annotated metabolites are presented in supplementary data Supplementary Tables S4, S5. Annotated metabolites indicated as important for the differences between the sampling techniques could be divided into three general categories; 1) small hydrophilic metabolites, 2) lipids and 3) peptides, and the following discussion will focus on those three general metabolite categories.

### Differences in the Metabolite Profiles

#### Small Hydrophilic Metabolites

**FIGURE 6:**
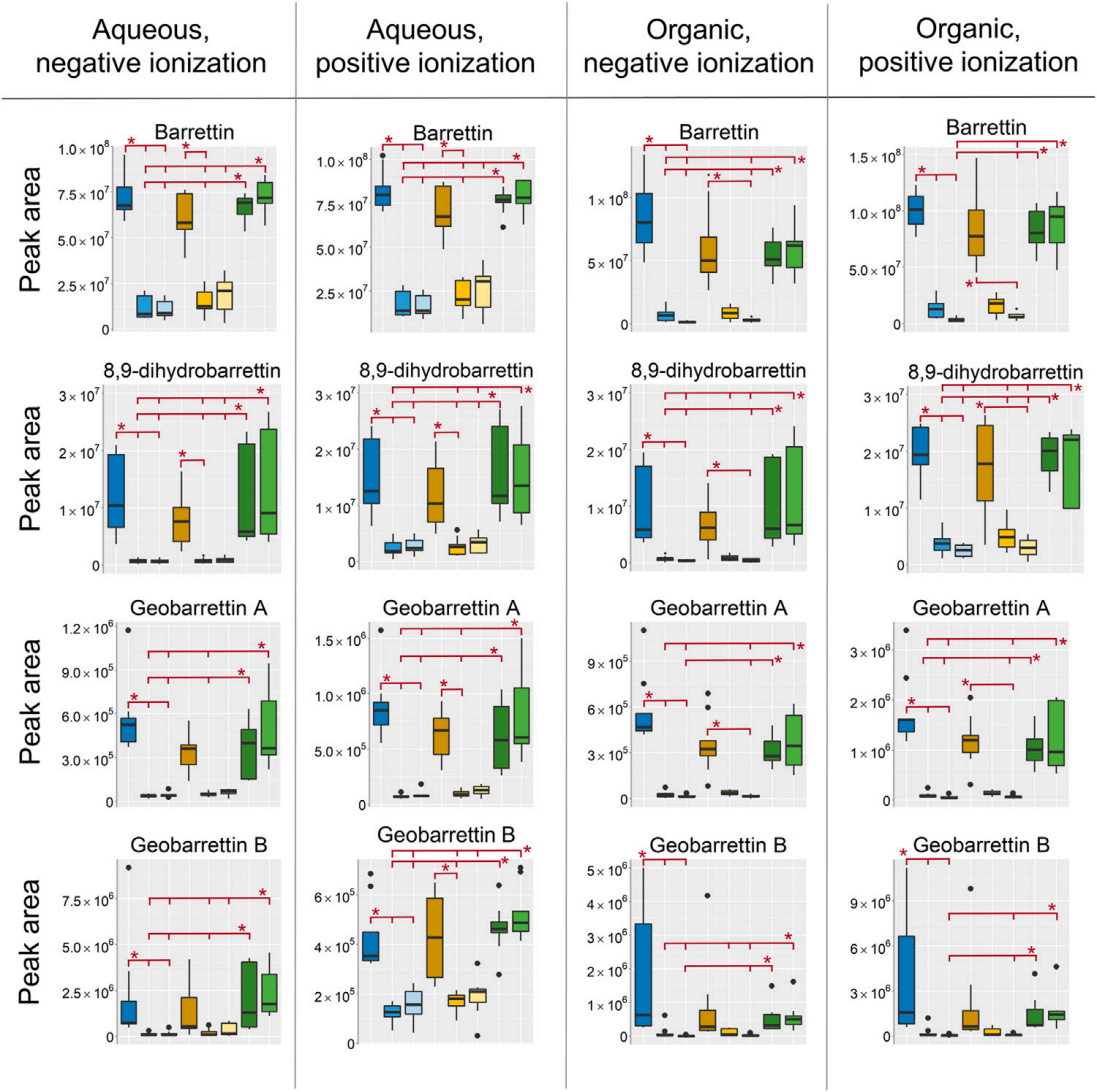
Box-plots of peak areas (*y*-axis) of barrettin, 8,9-dihydrobarrettin, geobarrettin A and geobarrettin B, top to bottom row, in the four respective datasets, from left to right. EtOH extr.1 (dark blue 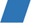), EtOH extr.2 (blue 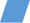), EtOH sp. (light blue 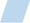), MeOH extr.1 (dark orange 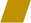), MeOH extr.2 (yellow 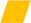), MeOH sp. (light yellow 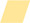), frozen (dark green 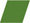), and flash frozen (green 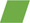). Statistical significance (*p* < 0.05) is marked by * in the respective box-plots, all *p*-values from the Kruskal Wallis and Nemenyi post-hoc testing can be found in supplementary data, Supplementary Tables S6–S9.

Small hydrophilic metabolites (Mw < 600) were generally found in the aqueous extracts analyzed in HILIC as was expected. However, some of the most well-known secondary metabolites from *Geodia barretti* such as barettin and derivatives thereof, which have both hydrophilic and hydrophobic properties, were extracted in both the aqueous and the organic extracts (Figure 6). The barettins and geobarettins A-B were found in both positive and negative ionization mode with some exceptions, such as the L-6-bromohypaphorine, geobarettin C, 6-bromoconicamin, and 8-hydroxy-6-bromoconicamin, which were only detected in positive mode as they are permanently positively charged due to a quaternary ammonium group. However, as discussed under *Principal Component Analysis*, the biological variation was particularly high for L-6-bromohypaphorine, geobarettin C, 6-bromoconicamin and 8-hydroxy-6-bromoconicamin with extreme intensities in specimen 1 that could not be related to differences in extraction, sampling or storage. They will therefore not be further included in the discussion on extraction and sampling conditions. All annotated metabolites from all analysis modes can be found in the supplementary data, Supplementary Tables S4, S5.

In all of the extracts the highest intensity of the bromoindole derivatives were found in the EtOH extr.1, MeOH extr.1, frozen and the flash frozen samples. The EtOH and MeOH secondary extracts and sponges were the sample groups with the lowest intensity, suggesting that a majority of the metabolites were extracted by the first round of solvents. However, there were no large differences between the MeOH and EtOH samples, the intensity was slightly higher in the EtOH extracts than in the MeOH extracts for barettin, 8,9-dihydrobarettin and geobarettin A but not for geobarettin B (not significant).

**FIGURE 7:**
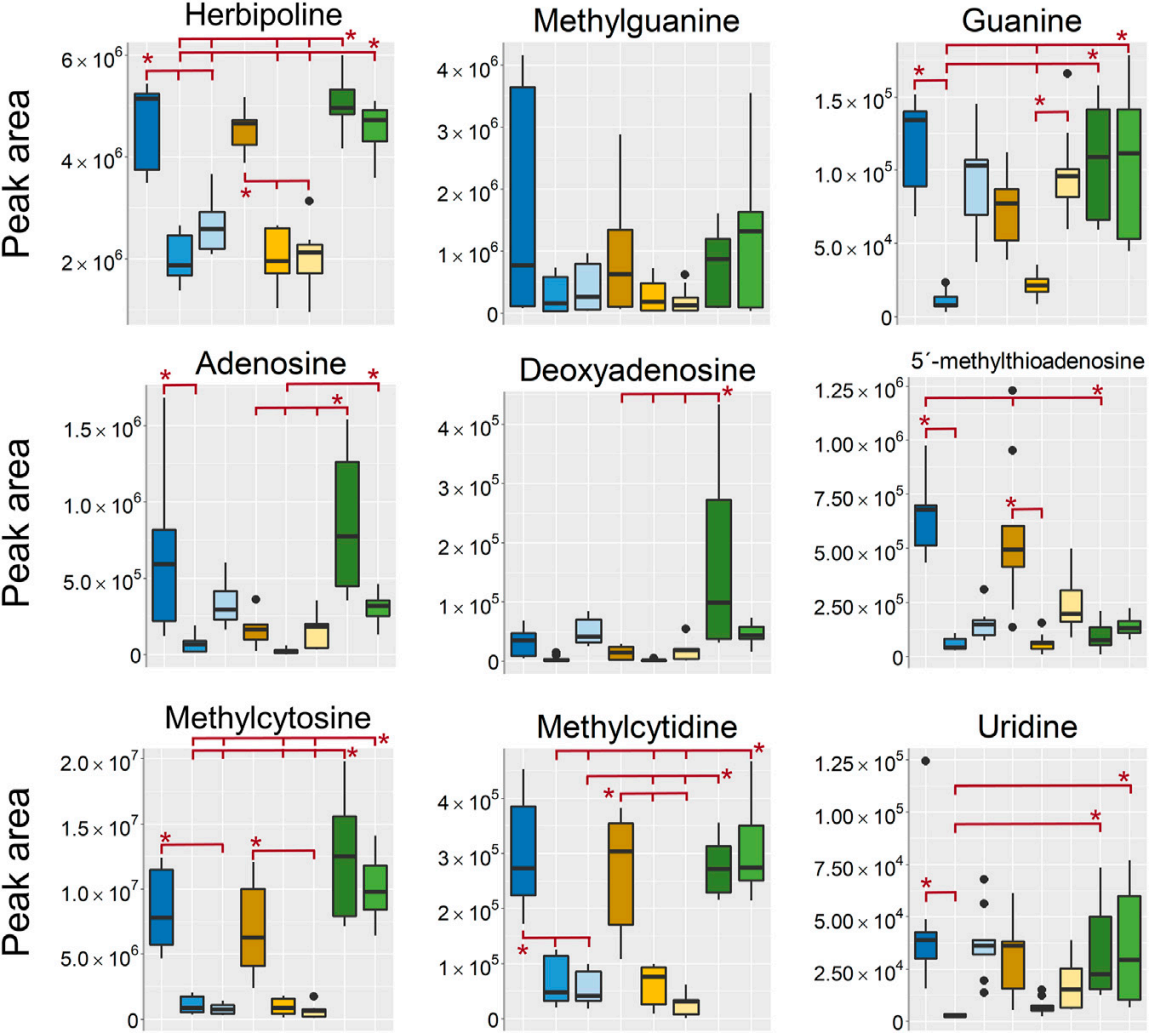
Boxplots of peak areas (*y*-axis) of nucleotides, nucleosides and nucleobases found as differentiating between the sample treatments in the aqueous extracts. All boxplots in the figure was based on data from the analysis in positive ionization mode, except for uridine which, was detected in negative ionization mode. EtOH extr.1 (dark blue 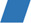), EtOH extr.2 (blue 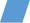), EtOH sp. (light blue 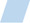), MeOH extr.1 (dark orange 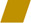), MeOH extr.2 (yellow 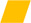), MeOH sp. (light yellow 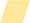), frozen (dark green 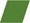), and flash frozen (green 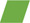). Outliers are denoted as dots in the plots. Statistical significance (*p* < 0.05) is marked by * in the respective box-plots, all *p*-values from the Kruskal Wallis and Nemenyi post-hoc testing can be found in supplementary data, Supplementary Table S10.

The rest of the small hydrophilic metabolites that were indicated as differentiating between the sample treatments in the OPLS-DA models were only detected in the aqueous extracts in the HILIC analyses. A group of metabolites that was found in both negative and positive ionization was nucleotides, nucleosides and nucleobases (Figure 7). In line with the results from the barettin metabolites, the nucleotides were found in highest intensity in the EtOH/MeOH extr.1 and the frozen/flash frozen samples however, with some variations. The two adenine based nucleosides adenosine and deoxyadenosine were interestingly found in higher intensity in the frozen samples than in the flash frozen (not significantly), however, the technical variation in the frozen group was much higher than in the flash frozen group. That could indicate an increased breakdown of adenine based nucleotides, e.g., ATP, to its respective nucleosides in the frozen samples as compared to the flash frozen, which would be in line with previous results where flash freezing is generally recommended for metabolomics studies to quench the metabolism as quickly as possible after sample collection to avoid enzymatic degradation or alterations of the metabolites post-sampling (Kim and Verpoorte, 2010; Dudzik et al., 2018; González-Riano et al., 2020; Salem et al., 2020).

The nucleotides, nucleosides and nucleobases, except for methylcytidine and methylcytosine, were found in relatively high intensity in the EtOH/MeOH sponges, in some cases in as high intensity as in the frozen/flash frozen sponges. This suggests that some metabolites were more efficiently extracted if the sponge samples were freeze-dried and mechanically processed as compared to just extracted in EtOH/MeOH directly. One of the few metabolites that was different between the extraction solvents and the frozen sponges was 5′-deoxy-5′-methyltioadenosine, which, was found in higher intensity in the EtOH/MeOH extr.1 than in the frozen/flash frozen sponges. This substance is not dramatically different either in polarity or structure from the other nucleosides/nucleotides which, were in high intensity in the frozen samples. However, it is possible that it is related to where 5′-deoxy-5′-methyltioadenosine is produced and located in the cells and that is readily extracted from the fresh sponge.

**FIGURE 8:**
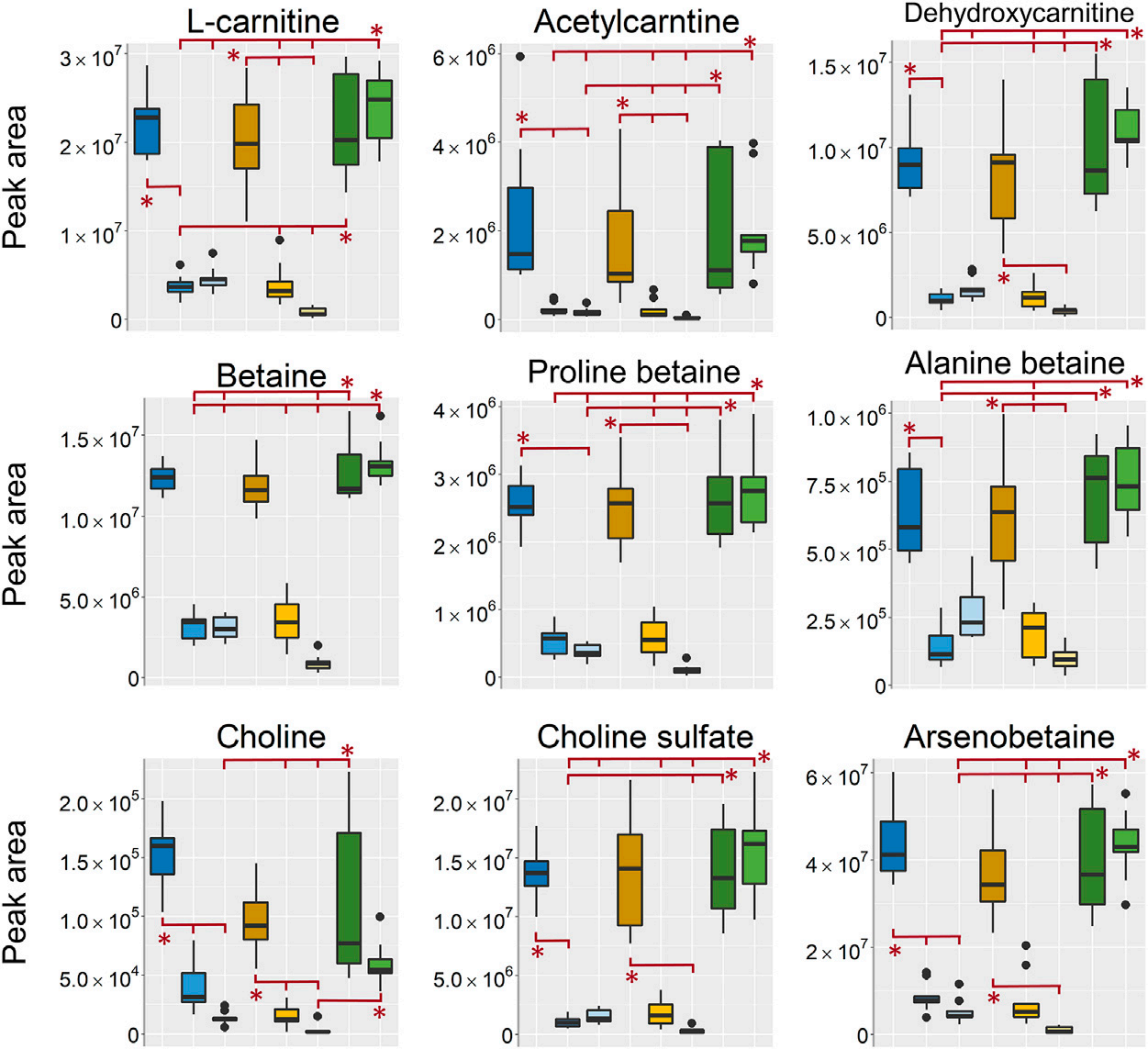
Boxplots of peak areas (y-axis) of betaines, carnitines and other osmolytes found as differentiating between the sample treatments. All boxplots in the figure was based on data from the analysis in positive ionization mode. EtOH extr.1 (dark blue 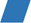), EtOH extr.2 (blue 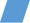), EtOH sp. (light blue 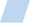), MeOH extr.1 (dark orange 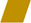), MeOH extr.2 (yellow 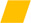), MeOH sp. (light yellow 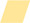), frozen (dark green 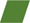) and flash frozen (green 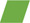). Outliers are denoted as dots in the plots. Statistical significance (p < 0.05) is marked by * in the respective box-plots, all p-values from the Kruskal Wallis and Nemenyi post-hoc testing can be found in supplementary data, Supplementary Table S11.

In the aqueous extracts, analyzed in positive ionization mode a large group of metabolites found as differentiating between sampling groups were different carnitines, betaines and other types of quaternary ammonium compounds (Figure 8). In line with previously discussed metabolites the intensity was highest in the EtOH/MeOH extr.1 and the frozen/flash frozen samples. The exception being choline that was found in highest intensity in the EtOH extr.1 followed by MeOH extr.1, frozen and flash frozen. Its higher intensity in EtOH extr.1 (and MeOH extr.1) could be related to extraction of phosphatidylcholine lipids and subsequent hydrolysis of the phosphatidylcholines, which, were found in higher intensity in the aqueous extracts of EtOH/MeOH extr.1 than in the frozen samples (discussed in *Peptides*).

In conclusion for extraction of small hydrophilic metabolites, the aqueous extracts of 1:1 MeOH:H_2_O, analyzed with HILIC-ESI-MS proved the most valuable and informative. The highest signal intensities were observed in the EtOH/MeOH extr.1 and the frozen/flash frozen samples. Regarding the samples where the sponges were stored in either EtOH or MeOH, most metabolites could be extracted from the sponge pieces that had been stored in EtOH or MeOH, however, in much lower intensity as compared to the original extracts. Hence, if samples have been stored in solvent, the solvent should not be thrown away as the solvents may likely contain most metabolites. Moreover, it is relevant for researchers to know that they could still extract most small hydrophilic metabolites, albeit in low amounts, from a sponge museum sample, which has been originally fixed in EtOH (the usual case currently). There were no large differences between the frozen and flash frozen samples, however, the technical variation was lower in the flash frozen samples and there were indications of less metabolite degradation in the flash frozen samples, e.g., nucleotides.

#### Lipids

**FIGURE 9:**
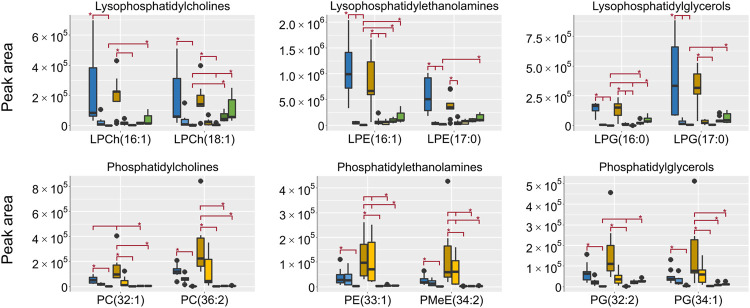
Box plots of peak areas (*y*-axis) of lipids found as differentiating between the sample groups in the aqueous extracts, the data was collected in HILIC negative ionization. Presented here are examples from each of the lipid classes, all annotated lipids can be found in the supplementary data. EtOH extr.1 (dark blue 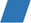), EtOH extr.2 (blue 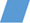), EtOH sp. (light blue 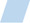), MeOH extr.1 (dark orange 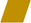), MeOH extr.2 (yellow 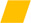), MeOH sp. (light yellow 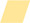), frozen (dark green 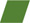), and flash frozen (green 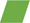). Outliers are denoted as dots in the plots. Statistical significance (*p* < 0.05) is marked by * in the respective box-plots, all *p*-values from the Kruskal Wallis and Nemenyi post-hoc testing can be found in supplementary data, Supplementary Table S12.

**FIGURE 10:**
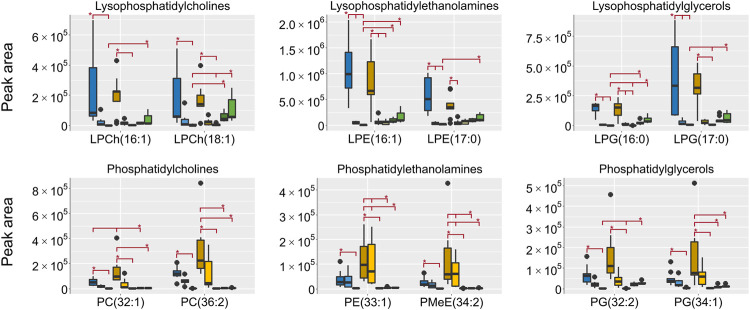
Box plots of peak areas (*y*-axis) of lipids found as differentiating between the sample groups in the organic extracts, the data was collected in RP negative ionization. Presented here are examples from each of the lipid classes, all annotated lipids can be found in the supplementary data. EtOH extr.1 (dark blue 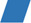), EtOH extr.2 (blue 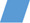), EtOH sp. (light blue 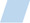), MeOH extr.1 (dark orange 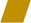), MeOH extr.2 (yellow 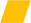), MeOH sp. (light yellow 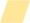), frozen (dark green 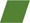), and flash frozen (green 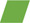). Outliers are denoted as dots in the plots. Statistical significance (*p* < 0.05) is marked by * in the respective box-plots, all *p*-values from the Kruskal Wallis and Nemenyi post-hoc testing can be found in supplementary data, Supplementary Table S13.

All lipids were annotated based on accurate mass and when possible fragmentation was compared to reference spectra, however, no investigation was made to determine the exact fatty acid composition. The focus was to determine the respective lipid classes to be able to draw general conclusions on the differences in metabolome composition depending on the sampling. In HILIC, lipids are generally not well retained, however, lipids with polar groups such as the phosphatidylcholines (PCh) are retained. In the aqueous extracts the lipid classes lysophosphatidylcholines (LPCh), lysophosphatidylethanolamines (LPE) and lysophosphatidylglycerols (LPG) were all found in high intensities in both the EtOH extr.1 and the MeOH extr.1 (Figure 9). The regular phospholipids such as the PCh:s, the phosphatidylethanolamines (PE) and phosphatidylglycerols (PG) were all found in high intensity in the MeOH extr.1 and extr.2, particularly the PE:s were found in high intensity in the MeOH extr.2 samples. The intensities of lipids in the frozen and flash frozen samples were lower than in the EtOH and MeOH extracts, especially regarding the PCh:s, PE:s and PG:s. Phospholipids together with inorganic ions are some of the main components causing matrix effects and particularly ion suppression in HILIC analyses of polar metabolites (Mess et al., 2009; Tan et al., 2012; Ekdahl et al., 2013; Elmsjö et al., 2018; Erngren et al., 2019). To avoid matrix effects and ion suppression in the analysis of small polar metabolites, the main interest in the HILIC analysis, it is preferable with as low phospholipid concentration as possible, especially as the analysis in reversed phase is much more suitable for the analysis of lipids. Therefore, in that regard, the frozen or flash frozen sample treatments would be preferred due to the lower relative abundance of lipids in the aqueous extracts. In the organic extracts high intensities for all the investigated lipid classes were found in the MeOH extracts, and especially high intensities were found for PE:s and PG:s in the MeOH extr.2 samples. Interestingly the MeOH seem more efficient at extracting lipids from the sponge pieces as compared to the EtOH (Figures 9, 10). In the frozen and flash frozen samples the relative abundances of lipids were higher in the organic extracts than in the aqueous extracts, the intensity was in the same range as for the MeOH extracts for the LPCh:s, LPE:s, and LPG:s, however, much lower intensity was observed for the PE:s and PG:s (Figure 10). The lysophospholipids seem to be extracted to a large extent to the EtOH and MeOH that the sponges were stored in, as very low intensities of the lysophospholipids were detected in the EtOH/MeOH sp. samples, much lower than in the frozen/flash frozen samples. However, the PE:s and PG:s were found in the same intensity range in all the sponge samples, EtOH/MeOH sp. and frozen/flash frozen indicating that they were not extracted to the EtOH/MeOH to the same extent (Figure 10).

The fact that MeOH extracted more phospholipids, particularly the more hydrophobic phospholipids e.g., the PCh:s, PG:s, and PE:s, seems a bit counterintuitive as the ethanol is more hydrophobic and also more efficient in disrupting cell membranes as compared to methanol (Gustafson and Tagesson, 1985; McKarns et al., 1997). However, it is possible that the methanol is able to disrupt the extracellular matrix (mesohyl) in the sponges more efficiently and thereby extract more lipids. Methanol at high concentrations have been reported to destabilize collagen more efficiently than ethanol and the extracellular matrix in sponges consists to a large degree of different types of collagen (Bianchi et al., 1970; Ehrlich et al., 2018). The destabilization of the extracellular matrix could lead to more efficient extraction of lipids within the matrix or alternatively that the methanol is able to penetrate the sponge pieces more efficiently and thereby extract more phospholipids.

The phospholipids were readily extracted by EtOH and MeOH and particularly to the MeOH. In the aqueous extracts of especially the EtOH/MeOH extr.1, high concentrations of lysophospholipids were found. The lysophospholipids are known to cause matrix effects on co-eluting analytes and as the main focus in the aqueous extracts were the small hydrophilic metabolites, minimal concentrations of lysophospholipids are preferred. Consequently, if the metabolites are fractioned in an aqueous and an organic extract as in this case, the frozen or flash frozen sampling is preferred as a minimal amount of phospholipids were extracted to the aqueous extracts. However, they could be analyzed in the organic extracts.

#### Peptides

**FIGURE 11:**
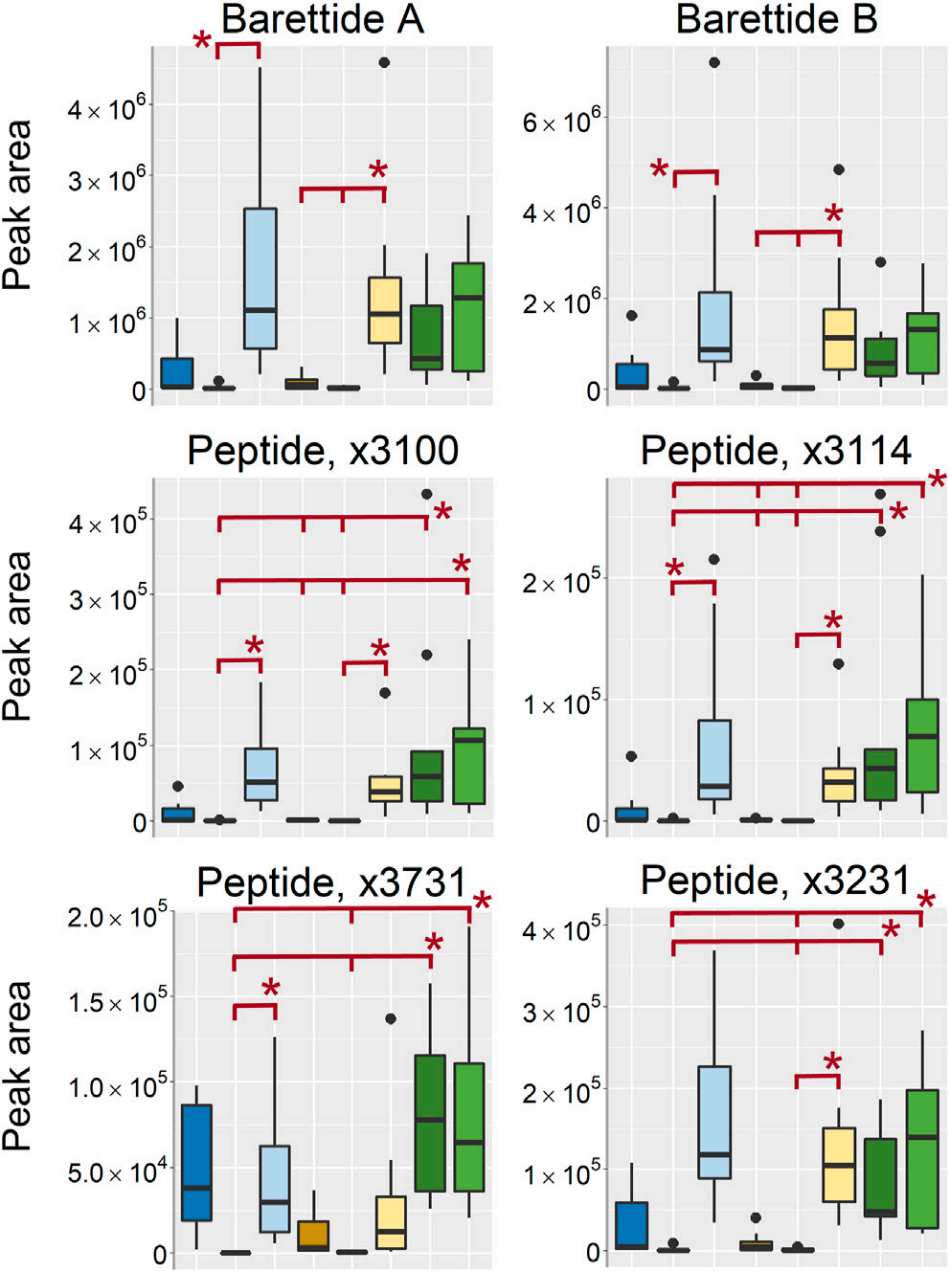
Boxplots of peptides found as differentiating between the sample treatments. All boxplots in the figure was based on data from the analysis of organic extracts in positive ionization mode. EtOH extr.1 (dark blue 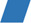), EtOH extr.2 (blue 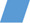), EtOH sp. (light blue 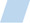), MeOH extr.1 (dark orange 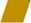), MeOH extr.2 (yellow 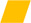), MeOH sp. (light yellow 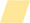), frozen (dark green 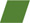), and flash frozen (green 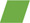). Outliers are denoted as dots in the plots. Statistical significance (*p* < 0.05) is marked by * in the respective box-plots, all *p*-values from the Kruskal Wallis and Nemenyi post-hoc testing can be found in supplementary data, Supplementary Table S14.

Peptides of the molecular mass of 3000–4000 u is usually not included in the metabolome, however, as the peptide signal intensities were indicated as some of the most important differences between the sampling conditions and the peptides often are of interest for natural products research they were included in the discussion. A few previously known and reported peptides in *G. barretti* such as the barettides A and B as well as five previously unreported peptides were found in the organic extracts (Figure 11). They were among the substances that were found in higher intensity in the sponges stored in EtOH/MeOH and the frozen/flash frozen samples than in the EtOH/MeOH extracts. However, there was one exception, the peptide named X3732 detected as [M+3H]^3+^ at *m/z* 1243.5203 (monoisotopic peak), that was found in high intensity in the EtOH extr.1 samples as well. The fact that the peptides were found in highest intensity in the sponge pieces (EtOH/MeOH sp. and frozen/flash frozen) suggest that their extraction may benefit from the freeze-drying and mechanical processing of the freeze-dried matter.

## Method Considerations and Recommendations

For a wide coverage of the sponge metabolome and the possibility to analyze anything from small hydrophilic metabolites and lipids to peptides with low technical variability and high precision in relative quantifications, flash freezing directly after sampling proved to be the most reliable option. The flash frozen, and to some extent the frozen sample group, were not significantly higher in intensity as compared to the EtOH extr.1 and MeOH ext.1. However, a vast majority of the investigated metabolites could be detected in high intensity with low variation in the flash frozen samples. Moreover, in the flash frozen samples the extraction of lipids to the aqueous extracts were minimal, but efficient in the organic extracts, leading to an efficient separation between polar and non-polar metabolites during sample preparation. As phospholipids are known to cause ion-suppression of polar metabolites during HILIC analyses, the minimal extraction of them to the aqueous extracts can be considered a major benefit for the flash frozen and frozen sampling. If liquid nitrogen is not available during collection though, no large differences could be detected between the flash-frozen and frozen samples in this investigation. This suggests that the immediate quenching of the metabolism provided by the flash freezing might not have as large effects on the results as previously thought and that most of the metabolites discussed herein were stable and not prone to degradation post-sampling. However, there were indications of increased metabolite degradation in the frozen samples, especially of the adenine nucleotides. It should be noted though, that the samples were collected in Sweden in the end of March, the outside temperature was ∼10°C and the difference between the flash frozen and frozen group might be greater at higher temperatures. As there were indications of metabolite degradation in the frozen samples as compared to the flash-frozen samples among the metabolites investigated here, there are likely unstable metabolites not specifically targeted or picked up by the models here that would benefit from flash freezing. There were also indications of higher variation between replicates for the frozen samples as compared to the flash frozen samples, which could indicate that there were issues with metabolite degradation but that the time-frame studied here between sampling and metabolism quenching (45 min–2 h for the frozen samples) were not enough to detect any major increases or decreases in metabolite levels.

It should also be stressed that for untargeted or targeted metabolomics where the metabolites are quantitated either relatively or absolutely, the samples should be flash frozen or frozen. Fieldwork in remote areas may prevent freezing of samples and in that case, solvent fixation may be considered the next best solution. However, for high precision and accuracy of the quantitative response for samples fixated in solvent it is absolutely paramount that equally large pieces of sponge is fixated in equal volumes of solvent and that the samples are stored in equal conditions for at least similar periods of time, which probably is difficult to achieve with precision enough.

*G. barretti* mainly produce alkaloids and peptides as its specialized metabolites, which was reflected in the type of metabolites that was annotated and identified as well (Lidgren and Bohlin, 1986; Carstens et al., 2015; Olsen et al., 2016a; Di et al., 2018). This means that these results could likely be transferred to other sponge species with similar metabolomes and specialized metabolites. For species with completely different chemical classes as its main metabolites though, these results might not be directly transferrable or applicable. However, the high degree of extraction of many metabolites from the fresh sponges to the solvents and the depletion of many metabolites if the solvents are repeatedly exchanged and discarded can likely be generalized to most sponge species, as well as the discussion about quenching the metabolism immediately after sample collection to avoid metabolic degradation and alterations of the samples.

Most marine biologists currently preserve freshly collected animals in ethanol (70–96%) in order to preserve their tissues and DNA. Sponges are no exception with 96% ethanol as the standard fixative for optimal DNA preservation (Cárdenas, 2010). Since sponges release substantial amounts of water when they are immersed in ethanol, the percentage of ethanol tends to be reduced and therefore, to maintain the % of ethanol high, it is common to change the ethanol at least once and quite often twice some hours after collection. These first ethanol extract(s) are usually discarded. This study highlights to sponge biologists the importance of saving these first ethanol extracts, which non-invasively recover a large part of the sponge chemistry to be further used in studies of natural products, chemosystematics or metabolomics. This study also underlines to sponge researchers and chemists that ethanol-preserved specimens can still be used for chemistry studies, especially if the focus is large peptides and some lipids (even after several extractions). Our results should increase the value of museum collections, not only for the unique and diverse specimens they hold but also for the ethanol preserving these same specimens. Few studies so far have explored the potential of sponge museum specimens for chemistry studies: Lengger et al. (Lengger et al., 2017) have compared the sterol content of ethanol extracts and that of their respective sponge specimens and shown that in at least one out of three species the sterol content was comparable. Cárdenas (Cárdenas, 2016) has shown that ethanol extracts and their corresponding sponge specimens could be used for presence/absence studies of bromotyrosine derivatives. However, in the present study the long-term stability of metabolites or the abilities of the different sampling techniques to preserve the metabolome over longer time periods was not studied and would need to be validated.

## Conclusion

In conclusion, among the different sample treatments evaluated in the study, the flash frozen, frozen as well as the first ethanol and methanol extracts were the most metabolite rich and the sample treatments where most metabolites were found in high intensity. A majority of the metabolites were readily extracted to the solvents used for fixation of the sponge samples, therefore, the solvents used for fixation and storage of sponge samples should not be discarded. However, some lipid species and the larger peptides were not extracted to these solvents but could be extracted from the samples stored in solvent. The lipids and peptides were also extracted from the frozen samples. Freezing the samples either directly with liquid nitrogen or later proved the most versatile sampling method as most metabolites could be analyzed with high intensity, low technical variation and high precision regarding relative quantitation. Moreover, the most information rich and metabolite abundant extracts were the aqueous extracts analyzed with HILIC, with the exception of lipids and larger peptides which were found in the organic extracts analyzed with reversed phase.

## Data Availability

Metabolomics data have been deposited to the EMBL-EBI MetaboLights database (DOI: 10.1093/nar/gkz1019, PMID:31691833) with the identifier MTBLS2635.
