# Supplementary material for: The Effects of Sampling and Storage Conditions on the Metabolite Profile of the Marine Sponge Geodia barretti
Source: Front Chem. 2021 May 10;9:662659. doi: 10.3389/fchem.2021.662659 (PMC8141568; doi:10.3389/fchem.2021.662659)
Supplement: Supplementary file 1 [file Table1.DOCX]

**Supplementary data: The effects of sampling and storage conditions on the metabolome of marine sponge *Geodia barretti***

**Contents**

- Table S1 – Summary of pre-processing parameters
- Table S2 – Summary of features in all datasets
- Figure S1 – BPI Chromatograms AQ neg
- FigureS2 - BPI Chromatograms AQ pos
- Figure S3 – BPI Chromatograms ORG neg
- Figure S4 – BPI Chromatograms ORG pos
- Figure S5 – PCA loading plots from the aqueous extracts
- Figure S6 - PCA loading plots from the aqueous extracts
- Table S4 – Summary annotated metabolites in aqueous extracts
- Table S5 - Summary annotated metabolites in organic extracts
- Figure S5 – Bar-plots with all annotated lipids in aq, negative ionization
- Figure S6 – Bar-plots with all annotated lipids in org, negative ionization

*Table S1. Summary of all the parameters and values used for pre-processing in XCMS.*

| *Parameter* | *RPLC Negative* | *RPLC positive* | *HILIC Negative* | *HILIC positive* |
| --- | --- | --- | --- | --- |
| *Prefilter* | 1800 | 1500 | 1500 | 1500 |
| *No. of scans in prefilter* | 3 | 3 | 3 | 3 |
| *Noise* | 1200 | 1000 | 1000 | 1000 |
| *PPM* | 40 | 40 | 45 | 50 |
| *Band width (bw)* | 1.5 | 1.5 | 2 | 1.5 |
| *Peak width* | 10-55 | 10-55 | 10-65 | 10-65 |

*Table S2. Summary of the number of features after pre-processing in the respective analysis modes. The no. of features present in all QC samples and with a CV < 30 % (in the QC samples) was the number of features that was included for the multivariate data analysis. The calculations of CV % is based on non-scaled and non-transformed data.*

| *Analysis mode* | *Total no. of features after pre processing* | *No. of features with retention > 45 s* | *No. of features with CV < 30 % in the QC samples^1^* | *No. of features with CV < 15 % in the QC samples^1^* | *No. of features present in all QC, CV < 30 % ^2^* |
| --- | --- | --- | --- | --- | --- |
| Aq, negative ionization | 1697 | 1381 | 1326 (96 %) | 1061 (77 %) | 971 |
| Aq, positive ionization | 3552 | 2871 | 2802 (98 %) | 2379 (83 %) | 2138 |
| Org, negative ionization | 2606 | 2084 | 1944 (93 %) | 1310 (63 %) | 1560 |
| Org, positive ionization | 4264 | 3608 | 3154 (87 %) | 2085 (58 %) | 2522 |

*^1^Calculated as percent of the no. of features with retention > 45 s.
^2^The number of features included in the respective multivariate models.*


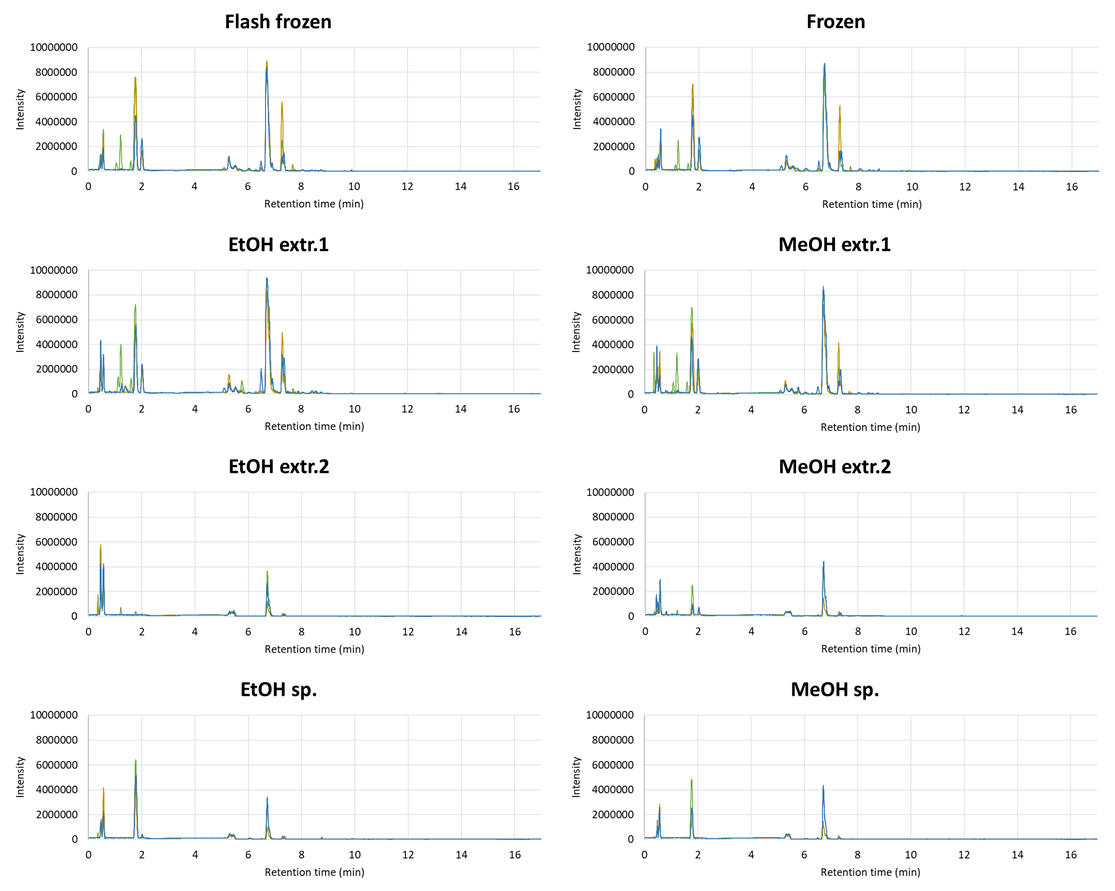

Figure S1. Examples of base peak intensity (BPI) chromatograms from the analysis of the aqueous extracts in negative ionization. One injection from each specimen is presented, specimen 1 – green line, specimen 2 – yellow line and specimen 3 – blue line.


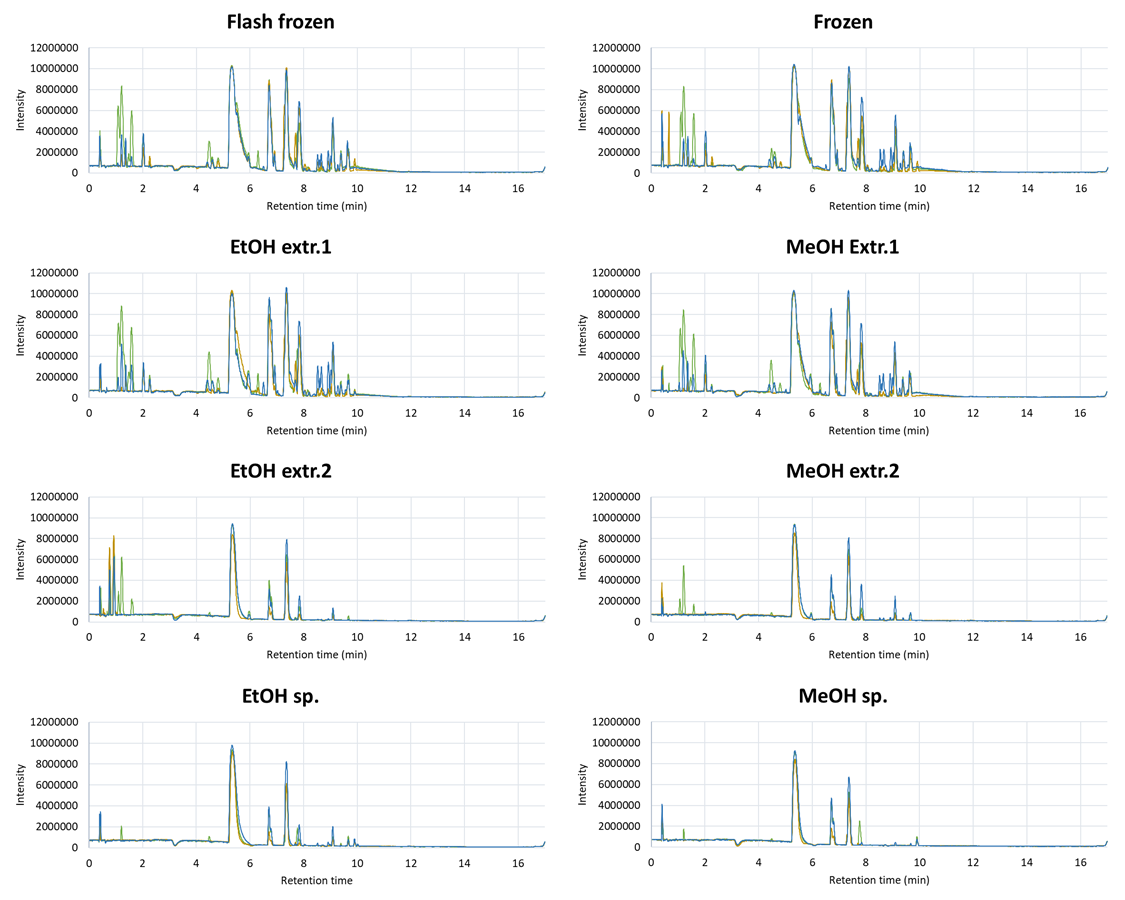

Figure S2. Examples of BPI chromatograms from the analysis of the aqueous extracts in positive ionization. One injection from each specimen is presented, specimen 1 – green line, specimen 2 – yellow line and specimen 3 – blue line.


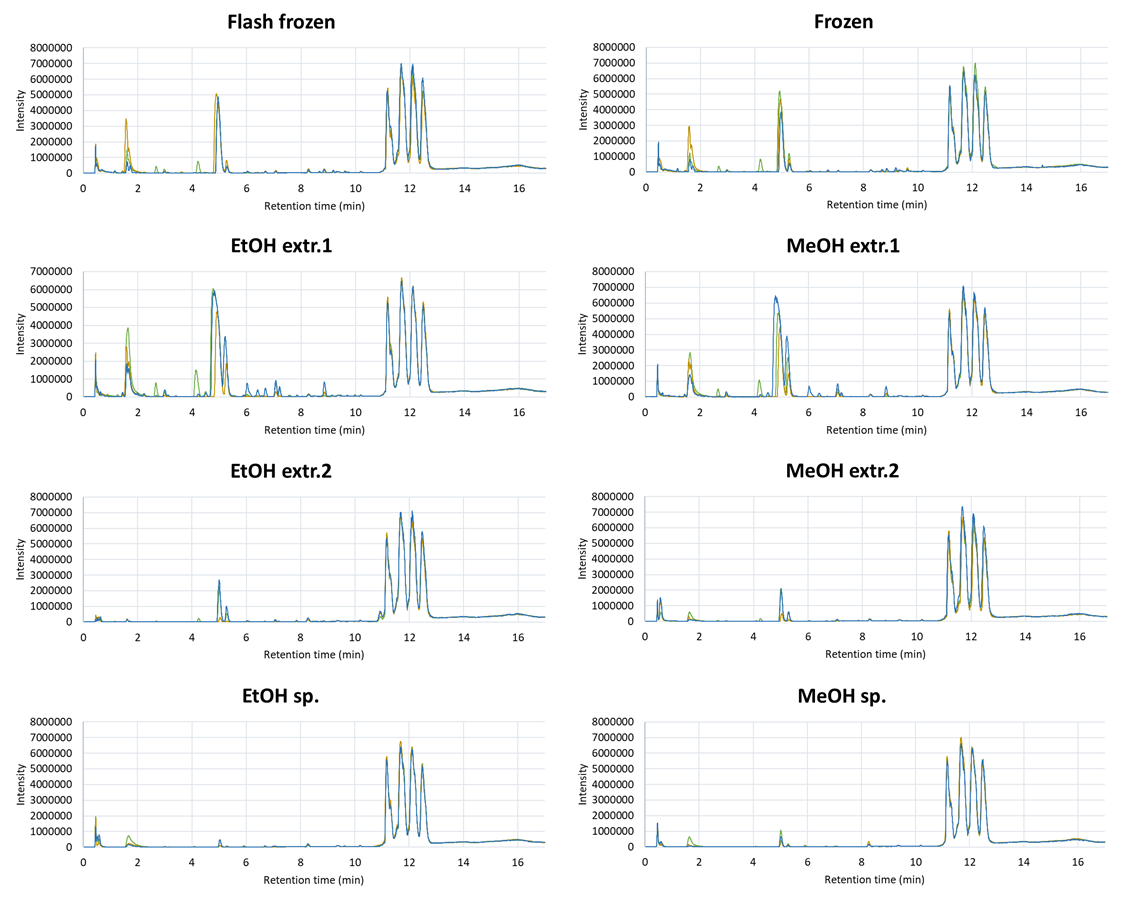

Figure S3. Examples of BPI chromatograms from the analysis of the organic extracts in negative ionization. One injection from each specimen is presented, specimen 1 – green line, specimen 2 – yellow line and specimen 3 – blue line.


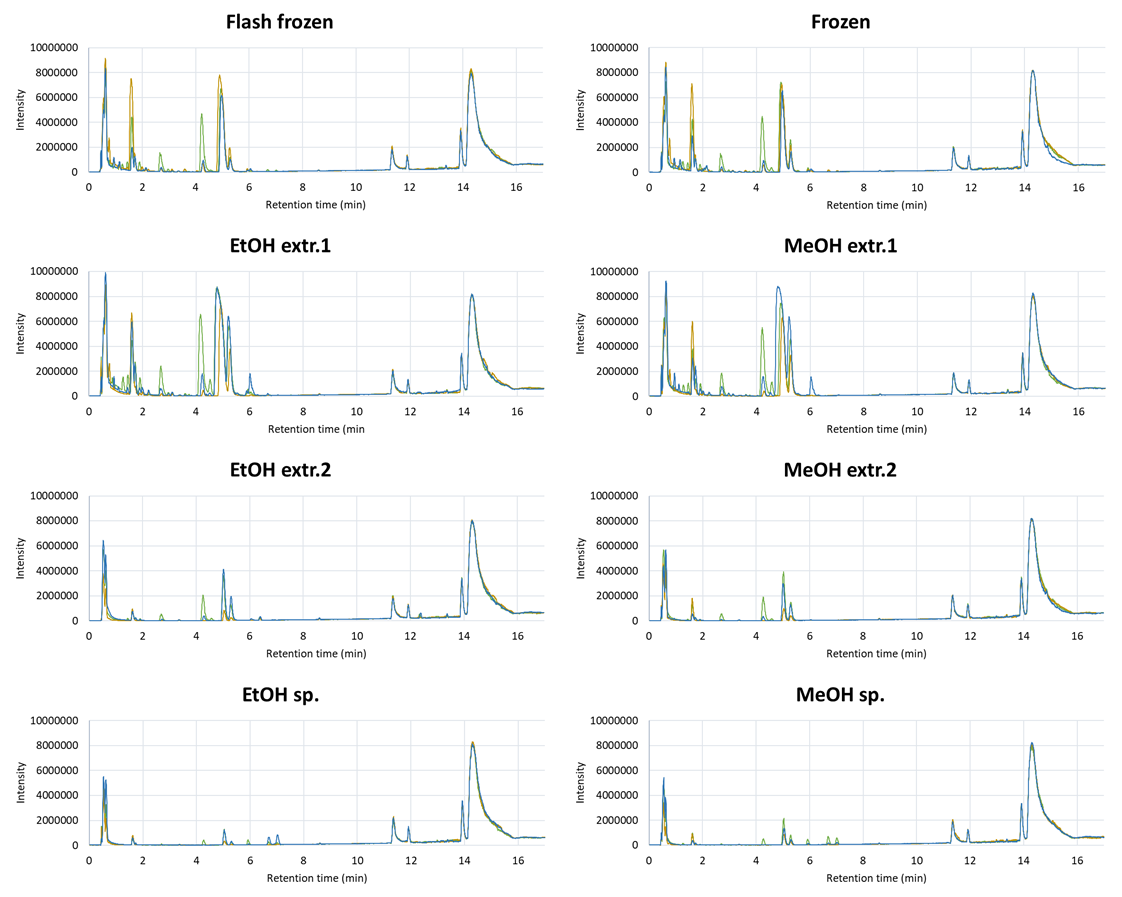


Figure S4. Examples of BPI chromatograms from the analysis of the organic extracts in positive ionization. One injection from each specimen is presented, specimen 1 – green line, specimen 2 – yellow line and specimen 3 – blue line.

Figure S5. PCA loadings plots from the aqueous extracts in negative ionization to the right and positive ionization to the left. The data points are colored based on their annotation and metabolite class.

Figure S6. PCA loadings plots from the organic extracts in negative ionization to the right and positive ionization to the left. The data points are colored based on their annotation and metabolite class.

*Table S4. Summary of all annotated metabolites in aqueous extracts, negative and positive ionization. For annotation Level 1-4 signifies the level of security regarding the identification/annotation according to Sumner et al.* (Sumner et al., 2007)*, level 1- compared to standard using MS/MS, retention time and accurate mass, level 2 – annotated substance based on accurate mass, fragmentation, comparisons to available reference data, elemental composition estimations, level 3 – annotated substance or substance class based on accurate mass, fragmentation or similar reference but with several possible candidates, level 4- accurate mass and elemental composition estimation but no known metabolites could be annotated.*

| ***Metabolite ID*** | ***Structure*** | ***Aqueous extracts, negative ionization*** | | | | ***Aqueous extracts, positive ionization*** | | | |
| --- | --- | --- | --- | --- | --- | --- | --- | --- | --- |
|  |  | ***Retention*** | ***m/z and adduct*** | ***Mass error*** | ***Annotation*** | ***Retention*** | ***m/z and adduct*** | ***Mass error*** | ***Annotation*** |
| Barettin |  | 6.73 min | [M-H]^-^ 417.067/419.065 | 1.14 ppm | Level 2 | 6.73 min | 419.085/421.083 [M+H]^+^ | 4.47 ppm | Level 2 |
| 8,9-dihydrobarettin |  | 7.29 min | 419.082/421.081 [M-H]^-^ | 2.57 ppm | Level 2 | 7.28 min | 421.098/423.097 [M+H]^+^ | 3.95 ppm | Level 2 |
| 8,9-dihydro-8-hydroxy-barettin |  |  |  |  |  | 7.71 min | 437.092/439.091 [M+H]^+^ | 3.95 ppm | Level 2 |
| Geobarettin A |  | 7.53 min | 449.055/451.054 [M-H]^-^ | 5.07 ppm | Level 1 | 7.57 min | 451.072/453.070 [M+H]^+^ | 2.05 ppm | Level 1 |
| Geobarettin B |  | 6.51 min | 415.051/417.049 [M-H]^-^ | 1.87 ppm | Level 1 | 6.51 min | 417.066/419.065 [M+H]^+^ | 3.42 ppm | Level 1 |
| Geobarettin C |  |  |  |  |  | 1.57 min | 295.044/297.045 [M+]^+^ | 0.34 ppm | Level 2 |
| 6-bromoconicamin |  |  |  |  |  | 1.21 min | 279.049/281.049 [M+]^+^ | 0.48 ppm | Level 2 |
| 8-hydroxy-6-bromoconicamin |  |  |  |  |  | 1.59 min | 297.055/299.057 [M+]^+^ |  | Level 2 |
| L-6-bromohypaphorine |  | 1.21 min | 323.037/325.035 [M-H]^-^ |  | Level 1 |  |  |  |  |
| X457 Unknown-Br |  | 7.77 min | 397.039/399.038 [Fragment]^-^ |  | Level 4 | 7.77 min | 458.129/460.128 |  | Level 4 |
| X310 Unknown-Br |  | 1.77 min | 309.903/311.902 |  | Level 4 | 1.79 min | 311.916/313.913 |  | Level 4 |
| Cyclo(Pro-Arg) |  | 7.35 min | 252.145 [M-H]^-^ | 4.26 ppm | Level 3 | 7.36 min | 254.165 [M+H]^+^ |  | Level 3 |
| Methylguanine | 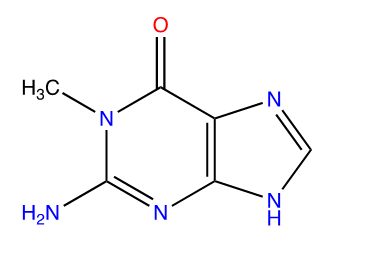 | 5.39 min | 164.056 [M-H]^-^ |  | Level 3 | 5.41 min | 166.073 [M+H]^+^ | 0.69 ppm | Level 3 |
| Herbipoline |  | 5.29 min | 178.072 [M-H]^-^ | 4.91 ppm | Level 3 | 5.33 min | 180.088 [M+H]^+^ | 2.92 ppm | Level 3 |
| Uridine |  | 2.38 min | 152.034 [Fragment]^-^ | 3.95 ppm | Level 1 |  |  |  | Level 1 |
| 5´-deoxy-5´methylthioadenosine |  |  |  |  |  | 1.08 min | 298.096 [M+H]^+^ | 4.65 ppm | Level 2 |
| Adenosine |  |  |  |  |  | 2.27 min | 268.103 [M+H]^+^ | 5.89 ppm | Level 1 |
| Deoxyadenosine | 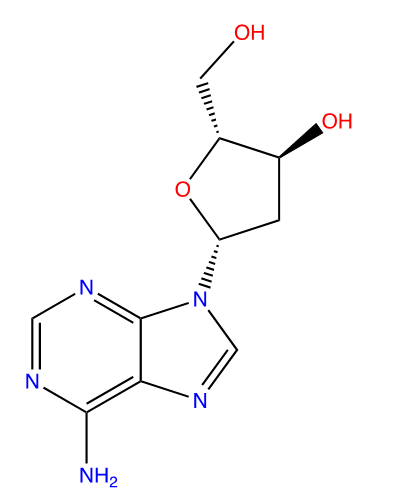 |  |  |  |  | 1.56 min | 252.108 [M+H]^+^ | 6.60 ppm | Level 2 |
| Guanine |  |  |  |  |  | 5.94 min | 152.057 [M+H]^+^ | 1.55 ppm | Level 1 |
| Methylcytidine | 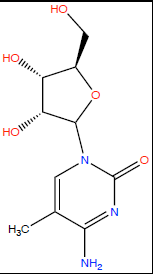 |  |  |  |  | 7.69 min | 258.108 [M+H]^+^ | 3.86 ppm | Level 3 |
| Methylcytosine |  |  |  |  |  | 4.59 min | 126.067 [M+H]^+^ | 2.09 ppm | Level 3 |
| X219, adenine derivative C10H13N5O |  |  |  |  |  | 8.98 min | 220.12 [M+H]^+^ |  | Level 4 |
| Histamine |  |  |  |  |  | 8.72 min | 112.087 [M+H]^+^ | 4.21 ppm | Level 1 |
| Histidinal | 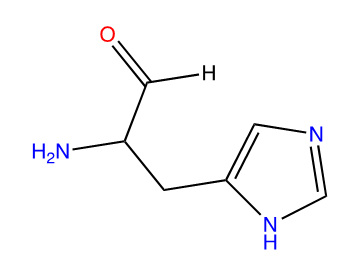 |  |  |  |  | 4.51 min | 140.082 [M+H]^+^ | 2.76 ppm | Level 2 |
| Trigonelline |  |  |  |  |  | 8.04 min | 138.056 [M+H]^+^ | 3.44 ppm | Level 3 |
| Arsenobetaine |  |  |  |  |  | 7.84 min | 179.009 [M+H]^+^ |  | Level 1 |
| 3-Carboxypropyl trimethylammonium (Dehydroxycarnitine) |  |  |  |  |  | 9.39 min | 146.119 [M+H]^+^ | 6.13 ppm | Level 3 |
| Acetylcarnitine |  |  |  |  |  | 8.59 min | 204.123 [M+H]^+^ | 2.86 ppm | Level 1 |
| Alaninebetaine |  |  |  |  |  | 9.67 min | 132.103 [M+H]^+^ | 4.13 ppm | Level 2 |
| Betaine |  |  |  |  |  | 7.45 min | 118.087 [M+H]^+^ | 1.66 ppm | Level 1 |
| Choline |  |  |  |  |  | 3.32 min | 104.107 [M]^+^ | 5.18 ppm | Level 1 |
| Cholinesulfate |  |  |  |  |  | 2.02 min | 184.064 [M+H]^+^ | 1.92 ppm | Level 2 |
| L-Carnitine |  |  |  |  |  | 9.09 min | 162.114 [M+H]^+^ | 6.06 ppm | Level 1 |
| Methyl-phosphorylcholine |  |  |  |  |  | 9.64 min | 198.089 [M+H]^+^ | 2.63 ppm | Level 3 |
| Prolinebetaine |  |  |  |  |  | 7.43 min | 144.103 [M+H]^+^ | 3.79 ppm | Level 2 |
| Taurine betaine |  |  |  |  |  | 5.70 min | 168.070 [M+H]^+^ | 3.34 ppm | Level 3 |
| Glycerophosphocholine |  |  |  |  |  | 10.41 min | 258.110 [M+H]^+^ | 2.51 ppm | Level 1 |

*Table S5. Summary of all annotated metabolites in organic extracts, negative and positive ionization. For annotation Level 1-4 signifies the level of security regarding the identification/annotation according to Sumner et al.* (Sumner et al., 2007)*, level 1- compared to standard using MS/MS, retention time and accurate mass, level 2 – annotated based on accurate mass, fragmentation, comparisons to available reference data, elemental composition estimations, level 3 – annotated based on accurate mass, fragmentation or similar reference but with several possible candidates, level 4- accurate mass and elemental composition estimation but no known metabolites could be annotated.*

| **Metabolite ID** | **Structure** | **Organic extracts, negative ionization** | | | | **Organic extracts, positive ionization** | | | |
| --- | --- | --- | --- | --- | --- | --- | --- | --- | --- |
|  |  | **Retention** | **m/z and adduct** | **Mass error** | **Annotation** | **Retention** | **m/z and adduct** | **Mass error** | **Annotation** |
| 8,9-dihydrobarrettin |  | 1.61 min | 419.082/421.080 [M+H]^-^ | 2.57 ppm | Level 2 | 1.62 min | 421.098/423.096 [M+H]^+^ | 1.72 ppm | Level 2 |
| 8,9-dihydro-8-hydroxy-barrettin |  |  |  |  |  | 1.80 min | 437.092/439.091 [M+H]^+^ | 3.95 ppm | Level 2 |
| Barrettin |  | 4.95 min | 417.067/419.0651 [M+H]^-^ | 1.14 ppm | Level 2 | 4.96 min | 419.084/421.081 [M+H]^+^ | 2.09 ppm | Level 2 |
| Geobarrettin A |  | 2.00 min | 449.055/451.0529 [M+H]^-^ | 5.07 ppm | Level 1 | 2.02 min | 451.072/453.070 [M+H]^+^ | 2.05 ppm | Level 1 |
| Geobarrettin B |  | 6.05 min | 415.050/417.490 [M+H]^-^ | 1.87 ppm | Level 1 | 6.05 min | 417.066/419.068 [M+H]^+^ | 3.42 ppm | Level 1 |
| Geobarrettin C |  |  |  |  |  | 5.22 min | 295.044/297.042 [M+]^+^ | 0.33 ppm | Level 1 |
| 6-bromoconicamin |  |  |  |  |  | 4.26 min | 279.049/281.047 [M+]^+^ | 0.48 ppm | Level 1 |
| 6-bromo-8-hydroxyconicamin |  |  |  |  |  | 2.70 min | 297.059/299.057 [M+]^+^ | 4.12 ppm | Level 1 |
| L-6-bromohypaphorine |  |  |  |  |  | 2.97 min | 325.055/327.052 [M+]^+^ | 0.38 ppm | Level 1 |
| X457 Unknown-Br |  | 1.26 min | 397.039/399.0381 [Fragment]^-^ |  |  | 1.27 min | 458.129/460.127 |  | Level 4 |
| X310 Unknown-Br |  | 1.67 min | 309.901/311.899 |  |  |  |  |  | Level 4 |
| Barrettide A |  | 6.69 min | 1070.08 [M+3H]^-^ |  | Level 3 | 6.70 min | 1072.100 [M+3H]^+^ |  | Level 3 |
| Barrettide B |  | 7.01 min | 1075.09 [M+3H]^-^ |  | Level 3 | 7.02 min | 1076.772 [M+3H]^+^ |  | Level 3 |
| Peptide X3732 |  | 5.93 min | 1242.51 [M+3H]^-^ |  | Level 3 |  |  |  | Level 3 |
| Peptide X3231 |  |  |  |  | Level 3 | 7.31 min | 1080.450 [M+3H]^+^ |  | Level 3 |
| Peptide X3100 |  |  |  |  | Level 3 | 6.49 min | 1034.085 [M+3H]^+^ |  | Level 3 |
| Peptide X3114 |  |  |  |  | Level 3 | 6.82 min | 1038.757 [M+3H]^+^ |  | Level 3 |
| Peptide X3286 |  |  |  |  | Level 3 | 6.60 min | 1096.104 [M+3H]^+^ |  | Level 3 |

***
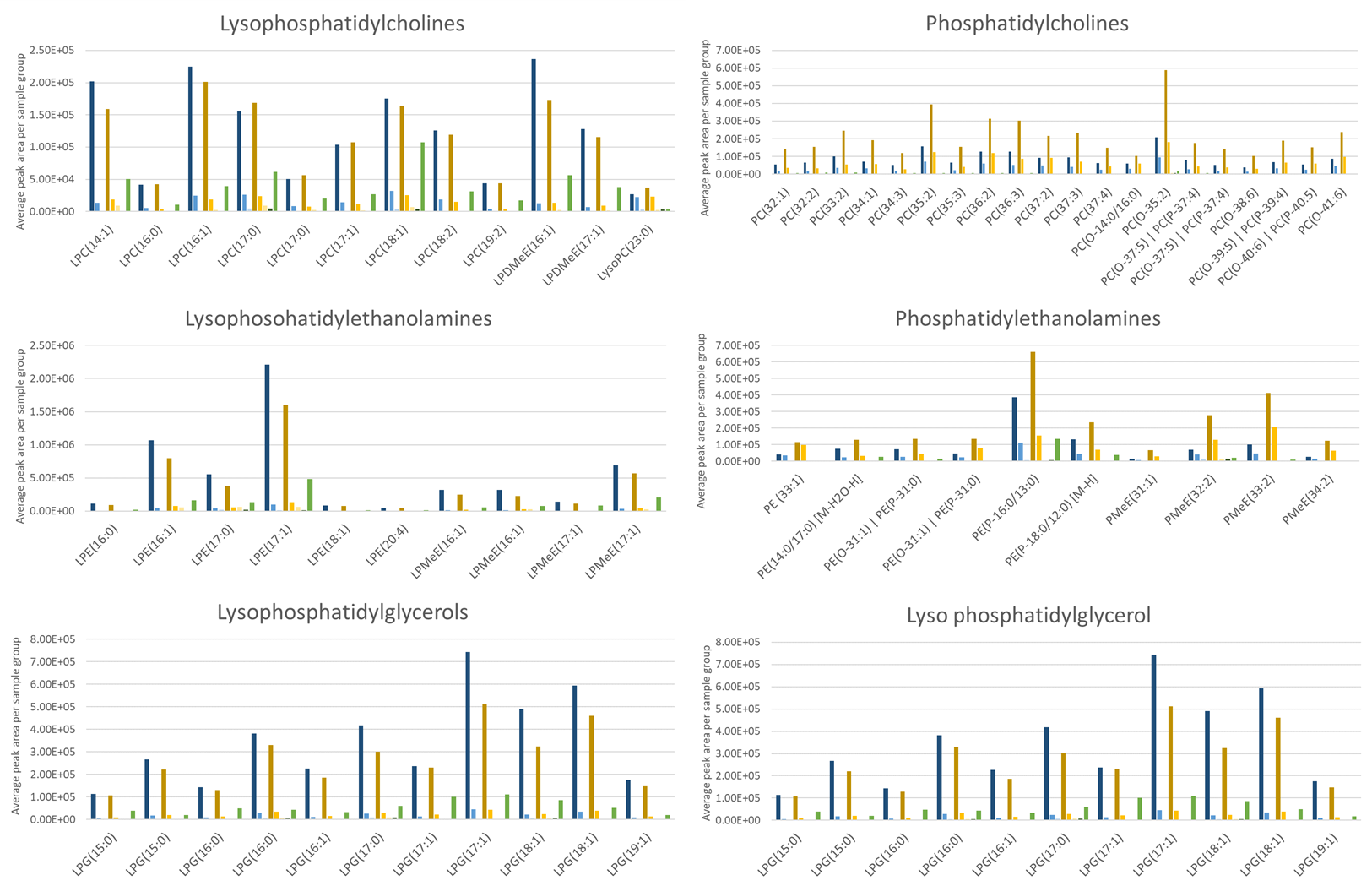
****Figure S5: Bar-plots with the average peak area per sample group on the y-axis of all annotated lipids in the aqueous extracts, in negative ionization.*

***
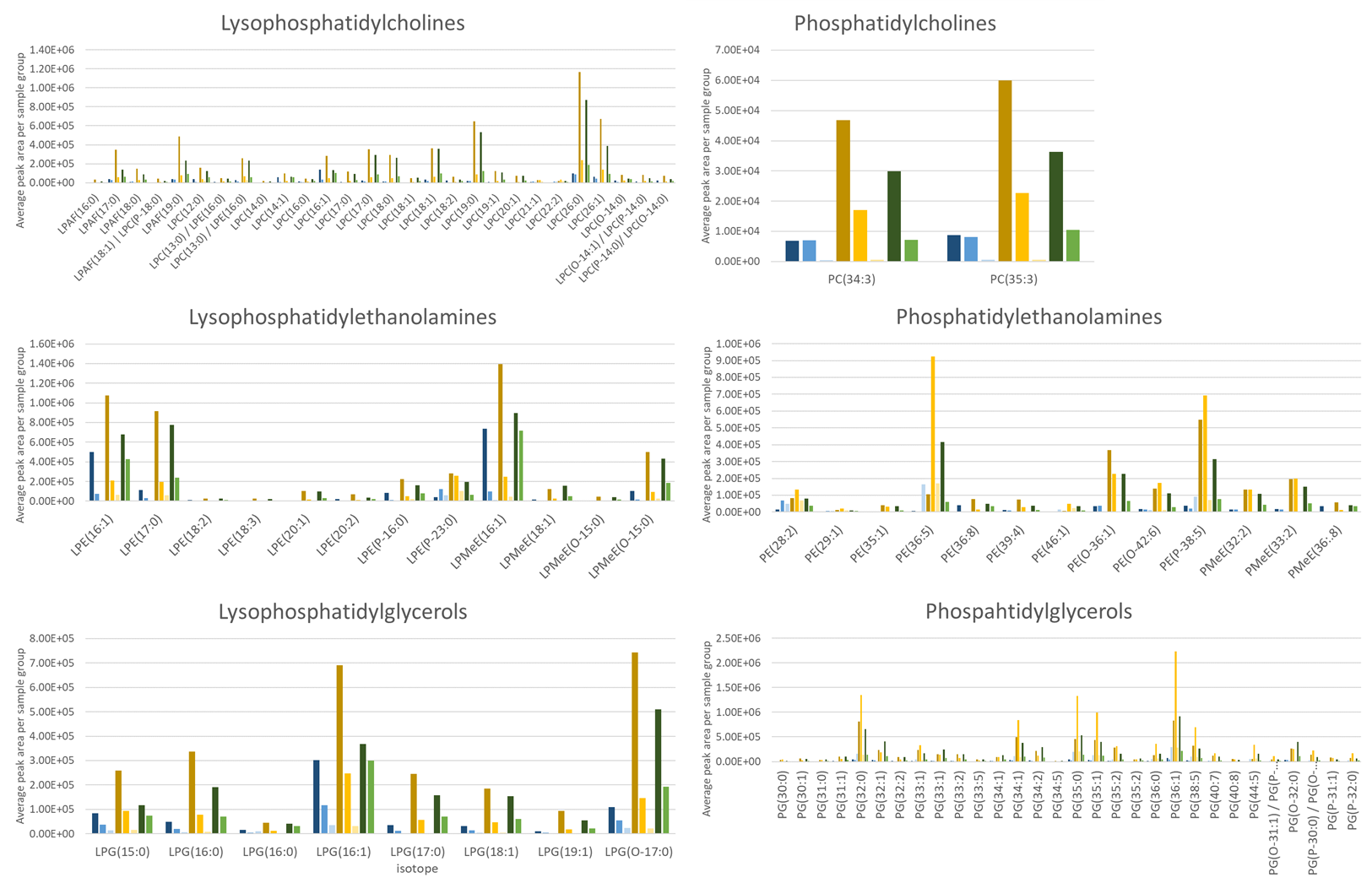
****Figure S6.* *Bar-plots with the average peak area per sample group on the y-axis of all annotated lipids in the organic extracts, in negative ionization.*

Table S6. Results from the Kruskal-Wallis test with Nemenyi post-hoc testing of the barrettin and similar metabolites in the aqueous extracts, analyzed in negative ionization.

| Barrettin, p= 1.278e-9 | | | | | | | |
| --- | --- | --- | --- | --- | --- | --- | --- |
|  | EtOH extr.1 | EtOH extr.2 | EtOH sp. | Frozen | Flash frozen | MeOH extr.1 | MeOH extr.2 |
| EtOH extr.2 | 0.00089 |  |  |  |  |  |  |
| EtOH sp. | 0.00052 | 1.00000 |  |  |  |  |  |
| Frozen | 0.99988 | 0.00498 | 0.00309 |  |  |  |  |
| Flash frozen | 1.00000 | 0.00035 | 0.00020 | 0.99828 |  |  |  |
| MeOH extr.1 | 0.99786 | 0.01178 | 0.00757 | 1.00000 | 0.98799 |  |  |
| MeOH extr.2 | 0.00438 | 0.99993 | 0.99958 | 0.02014 | 0.00189 | 0.04247 |  |
| MeOH sp. | 0.02090 | 0.99124 | 0.98017 | 0.07597 | 0.01005 | 0.13945 | 0.99986 |
| 8,9-dihydrobarrettin, p= 2.164e-9 | | | | | | | |
|  | EtOH extr.1 | EtOH extr.2 | EtOH sp. | Frozen | Flash frozen | MeOH extr.1 | MeOH extr.2 |
| EtOH extr.2 | 0.00237 |  |  |  |  |  |  |
| EtOH sp. | 0.00226 | 1.00000 |  |  |  |  |  |
| Frozen | 1.00000 | 0.00309 | 0.00296 |  |  |  |  |
| Flash frozen | 1.00000 | 0.00093 | 0.00089 | 0.99999 |  |  |  |
| MeOH extr.1 | 0.99983 | 0.01274 | 0.01225 | 0.99994 | 0.99761 |  |  |
| MeOH extr.2 | 0.00477 | 1.00000 | 1.00000 | 0.00615 | 0.00197 | 0.02334 |  |
| MeOH sp. | 0.01940 | 0.99916 | 0.99905 | 0.02421 | 0.00891 | 0.07597 | 0.99993 |
| Geobarrettin A, p=6.239e-10 | | | | | | | |
|  | EtOH extr.1 | EtOH extr.2 | EtOH sp. | Frozen | Flash frozen | MeOH extr.1 | MeOH extr.2 |
| EtOH extr.2 | 0.00003 |  |  |  |  |  |  |
| EtOH sp. | 0.00006 | 1.00000 |  |  |  |  |  |
| Frozen | 0.98500 | 0.00197 | 0.00353 |  |  |  |  |
| Flash frozen | 0.99863 | 0.00058 | 0.00108 | 0.99999 |  |  |  |
| MeOH extr.1 | 0.94589 | 0.00498 | 0.00855 | 1.00000 | 0.99951 |  |  |
| MeOH extr.2 | 0.00089 | 0.99642 | 0.99916 | 0.02699 | 0.01005 | 0.05537 |  |
| MeOH sp. | 0.00927 | 0.89248 | 0.94013 | 0.14326 | 0.06708 | 0.24170 | 0.99893 |
| Geobarrettin B, p=1.112e-7 | | | | | | | |
|  | EtOH extr.1 | EtOH extr.2 | EtOH sp. | Frozen | Flash frozen | MeOH extr.1 | MeOH extr.2 |
| EtOH extr.2 | 0.00520 |  |  |  |  |  |  |
| EtOH sp. | 0.01088 | 1.00000 |  |  |  |  |  |
| Frozen | 1.00000 | 0.00520 | 0.01088 |  |  |  |  |
| Flash frozen | 0.99523 | 0.00022 | 0.00055 | 0.99523 |  |  |  |
| MeOH extr.1 | 0.98272 | 0.10245 | 0.16775 | 0.98272 | 0.69432 |  |  |
| MeOH extr.2 | 0.02090 | 0.99993 | 1.00000 | 0.02090 | 0.00124 | 0.25294 |  |
| MeOH sp. | 0.23619 | 0.90100 | 0.95859 | 0.23619 | 0.03338 | 0.82082 | 0.98606 |

Table S7. Results from the Kruskal Wallis test with Nemenyi post-hoc testing of the barrettin and similar metabolites in the aqueous extracts, analyzed in positive ionization.

| Barrettin, p=1.228e-9 | | | | | | | |
| --- | --- | --- | --- | --- | --- | --- | --- |
|  | EtOH extr.1 | EtOH extr.2 | EtOH sp. | Frozen | Flash frozen | MeOH extr.1 | MeOH extr.2 |
| EtOH extr.2 | 0.00051 |  |  |  |  |  |  |
| EtOH sp. | 0.00025 | 1.00000 |  |  |  |  |  |
| Frozen | 0.99940 | 0.00477 | 0.00259 |  |  |  |  |
| Flash frozen | 1.00000 | 0.00062 | 0.00031 | 0.99966 |  |  |  |
| MeOH extr.1 | 0.99501 | 0.01046 | 0.00590 | 1.00000 | 0.99659 |  |  |
| MeOH extr.2 | 0.00289 | 0.99990 | 0.99916 | 0.02090 | 0.00345 | 0.04106 |  |
| MeOH sp. | 0.01637 | 0.98705 | 0.96709 | 0.08580 | 0.01904 | 0.14714 | 0.99977 |
| 8,9-dihydrobarrettin, p= 2.746e-9 | | | | | | | |
|  | EtOH extr.1 | EtOH extr.2 | EtOH sp. | Frozen | Flash frozen | MeOH extr.1 | MeOH extr.2 |
| EtOH extr.2 | 0.00193 |  |  |  |  |  |  |
| EtOH sp. | 0.00602 | 0.99999 |  |  |  |  |  |
| Frozen | 1.00000 | 0.00176 | 0.00554 |  |  |  |  |
| Flash frozen | 0.99999 | 0.00055 | 0.00189 | 0.99999 |  |  |  |
| MeOH extr.1 | 0.99982 | 0.01088 | 0.02899 | 0.99975 | 0.99523 |  |  |
| MeOH extr.2 | 0.00477 | 1.00000 | 1.00000 | 0.00438 | 0.00146 | 0.02377 |  |
| MeOH sp. | 0.02250 | 0.99774 | 0.99995 | 0.02090 | 0.00805 | 0.08710 | 0.99986 |
| Geobarrettin A, p= 6.41e-10 | | | | | | | |
|  | EtOH extr.1 | EtOH extr.2 | EtOH sp. | Frozen | Flash frozen | MeOH extr.1 | MeOH extr.2 |
| EtOH extr.2 | 0.00001 |  |  |  |  |  |  |
| EtOH sp. | 0.00037 | 0.99761 |  |  |  |  |  |
| Frozen | 0.98500 | 0.00103 | 0.01377 |  |  |  |  |
| Flash frozen | 0.99958 | 0.00018 | 0.00323 | 0.99993 |  |  |  |
| MeOH extr.1 | 0.98705 | 0.00093 | 0.01274 | 1.00000 | 0.99995 |  |  |
| MeOH extr.2 | 0.00119 | 0.98148 | 0.99999 | 0.03338 | 0.00891 | 0.03112 |  |
| MeOH sp. | 0.01225 | 0.77761 | 0.98799 | 0.17211 | 0.06297 | 0.16347 | 0.99879 |
| Geobarrettin B, p= 1.348e-9 | | | | | | | |
|  | EtOH extr.1 | EtOH extr.2 | EtOH sp. | Frozen | Flash frozen | MeOH extr.1 | MeOH extr.2 |
| EtOH extr.2 | 0.00164 |  |  |  |  |  |  |
| EtOH sp. | 0.03004 | 0.99318 |  |  |  |  |  |
| Frozen | 0.99977 | 0.00021 | 0.00590 |  |  |  |  |
| Flash frozen | 0.98606 | 0.00003 | 0.00108 | 0.99988 |  |  |  |
| MeOH extr.1 | 1.00000 | 0.00103 | 0.02090 | 0.99996 | 0.99375 |  |  |
| MeOH extr.2 | 0.03338 | 0.99124 | 1.00000 | 0.00668 | 0.00124 | 0.02334 |  |
| MeOH sp. | 0.15111 | 0.86431 | 0.99905 | 0.04247 | 0.01046 | 0.11489 | 0.99936 |

Table S8. Results from the Kruskal Wallis test with Nemenyi post-hoc testing of the barrettin and similar metabolites in the organic extracts, analyzed in negative ionization.

| Barrettin, p= 2.747e-10 | | | | | | | |
| --- | --- | --- | --- | --- | --- | --- | --- |
|  | EtOH extr.1 | EtOH extr.2 | EtOH sp. | Frozen | Flash frozen | MeOH extr.1 | MeOH extr.2 |
| EtOH extr.2 | 0.00259 |  |  |  |  |  |  |
| EtOH sp. | 0.00000 | 0.80286 |  |  |  |  |  |
| Frozen | 0.93863 | 0.11985 | 0.00065 |  |  |  |  |
| Flash frozen | 0.99624 | 0.03397 | 0.00008 | 0.99983 |  |  |  |
| MeOH extr.1 | 0.97424 | 0.07597 | 0.00030 | 1.00000 | 0.99999 |  |  |
| MeOH extr.2 | 0.00726 | 0.99999 | 0.63416 | 0.22280 | 0.07481 | 0.15111 |  |
| MeOH sp. | 0.00011 | 0.99642 | 0.99375 | 0.01404 | 0.00265 | 0.00757 | 0.97583 |
| 8,9-dihydrobarrettin, p= 3.769e-9 | | | | | | | |
|  | EtOH extr.1 | EtOH extr.2 | EtOH sp. | Frozen | Flash frozen | MeOH extr.1 | MeOH extr.2 |
| EtOH extr.2 | 0.00927 |  |  |  |  |  |  |
| EtOH sp. | 0.00031 | 0.99088 |  |  |  |  |  |
| Frozen | 1.00000 | 0.02292 | 0.00098 |  |  |  |  |
| Flash frozen | 1.00000 | 0.00985 | 0.00033 | 1.00000 |  |  |  |
| MeOH extr.1 | 0.99893 | 0.06297 | 0.00377 | 0.99998 | 0.99911 |  |  |
| MeOH extr.2 | 0.03705 | 0.99990 | 0.91485 | 0.07955 | 0.03901 | 0.18107 |  |
| MeOH sp. | 0.00133 | 0.99966 | 0.99998 | 0.00385 | 0.00143 | 0.01299 | 0.98446 |
| Geobarrettin A, p= 3.551e-10 | | | | | | | |
|  | EtOH extr.1 | EtOH extr.2 | EtOH sp. | Frozen | Flash frozen | MeOH extr.1 | MeOH extr.2 |
| EtOH extr.2 | 0.00060 |  |  |  |  |  |  |
| EtOH sp. | 0.00001 | 0.99193 |  |  |  |  |  |
| Frozen | 0.91103 | 0.06003 | 0.00377 |  |  |  |  |
| Flash frozen | 0.98017 | 0.02334 | 0.00108 | 0.99999 |  |  |  |
| MeOH extr.1 | 0.98554 | 0.01977 | 0.00087 | 0.99996 | 1.00000 |  |  |
| MeOH extr.2 | 0.01088 | 0.99605 | 0.77761 | 0.33025 | 0.17655 | 0.15720 |  |
| MeOH sp. | 0.00002 | 0.99706 | 1.00000 | 0.00602 | 0.00180 | 0.00146 | 0.84339 |
| Geobarrettin B, p= 1.179e-8 | | | | | | | |
|  | EtOH extr.1 | EtOH extr.2 | EtOH sp. | Frozen | Flash frozen | MeOH extr.1 | MeOH extr.2 |
| EtOH extr.2 | 0.01110 |  |  |  |  |  |  |
| EtOH sp. | 0.00004 | 0.88806 |  |  |  |  |  |
| Frozen | 0.99893 | 0.07253 | 0.00062 |  |  |  |  |
| Flash frozen | 1.00000 | 0.02250 | 0.00011 | 0.99994 |  |  |  |
| MeOH extr.1 | 0.97171 | 0.20490 | 0.00353 | 0.99989 | 0.99258 |  |  |
| MeOH extr.2 | 0.01834 | 1.00000 | 0.82082 | 0.10698 | 0.03579 | 0.27638 |  |
| MeOH sp. | 0.00360 | 0.99999 | 0.97082 | 0.02951 | 0.00788 | 0.09951 | 0.99983 |

Table S9. Table SX. Results from the Kruskal Wallis test with Nemenyi post-hoc testing of the barrettin and similar metabolites in the organic extracts, analyzed in positive ionization.

| Barrettin, p= 2.419e-10 | | | | | | | |
| --- | --- | --- | --- | --- | --- | --- | --- |
|  | EtOH extr.1 | EtOH extr.2 | EtOH sp. | Frozen | Flash frozen | MeOH extr.1 | MeOH extr.2 |
| EtOH extr.2 | 0.00283 |  |  |  |  |  |  |
| EtOH sp. | 0.00000 | 0.73025 |  |  |  |  |  |
| Frozen | 0.94863 | 0.11489 | 0.00035 |  |  |  |  |
| Flash frozen | 0.98272 | 0.06708 | 0.00014 | 1.00000 |  |  |  |
| MeOH extr.1 | 0.97879 | 0.07366 | 0.00016 | 1.00000 | 1.00000 |  |  |
| MeOH extr.2 | 0.00590 | 1.00000 | 0.60324 | 0.18107 | 0.11168 | 0.12154 |  |
| MeOH sp. | 0.00011 | 0.99566 | 0.98705 | 0.01225 | 0.00590 | 0.00668 | 0.98272 |
| 8,9-dihydrobarrettin, p= 3.266e-9 | | | | | | | |
|  | EtOH extr.1 | EtOH extr.2 | EtOH sp. | Frozen | Flash frozen | MeOH extr.1 | MeOH extr.2 |
| EtOH extr.2 | 0.02651 |  |  |  |  |  |  |
| EtOH sp. | 0.00051 | 0.96900 |  |  |  |  |  |
| Frozen | 1.00000 | 0.02951 | 0.00059 |  |  |  |  |
| Flash frozen | 1.00000 | 0.01178 | 0.00017 | 0.99999 |  |  |  |
| MeOH extr.1 | 0.99961 | 0.11489 | 0.00420 | 0.99975 | 0.99523 |  |  |
| MeOH extr.2 | 0.03901 | 1.00000 | 0.94306 | 0.04320 | 0.01800 | 0.15515 |  |
| MeOH sp. | 0.00211 | 0.99706 | 0.99998 | 0.00242 | 0.00077 | 0.01432 | 0.99193 |
| Geobarrettin A, p=3.865e-10 | | | | | | | |
|  | EtOH extr.1 | EtOH extr.2 | EtOH sp. | Frozen | Flash frozen | MeOH extr.1 | MeOH extr.2 |
| EtOH extr.2 | 0.00019 |  |  |  |  |  |  |
| EtOH sp. | 0.00001 | 0.99958 |  |  |  |  |  |
| Frozen | 0.86928 | 0.03835 | 0.00668 |  |  |  |  |
| Flash frozen | 0.97082 | 0.01225 | 0.00172 | 0.99997 |  |  |  |
| MeOH extr.1 | 0.94863 | 0.01800 | 0.00271 | 1.00000 | 1.00000 |  |  |
| MeOH extr.2 | 0.00668 | 0.98972 | 0.86928 | 0.31386 | 0.15111 | 0.19512 |  |
| MeOH sp. | 0.00002 | 0.99968 | 1.00000 | 0.00726 | 0.00189 | 0.00296 | 0.87888 |
| Geobarrettin B, p= 1.193e-8 | | | | | | | |
|  | EtOH extr.1 | EtOH extr.2 | EtOH sp. | Frozen | Flash frozen | MeOH extr.1 | MeOH extr.2 |
| EtOH extr.2 | 0.01178 |  |  |  |  |  |  |
| EtOH sp. | 0.00001 | 0.78406 |  |  |  |  |  |
| Frozen | 0.99050 | 0.14326 | 0.00077 |  |  |  |  |
| Flash frozen | 0.99963 | 0.05907 | 0.00017 | 0.99998 |  |  |  |
| MeOH extr.1 | 0.96709 | 0.22544 | 0.00180 | 1.00000 | 0.99944 |  |  |
| MeOH extr.2 | 0.01546 | 1.00000 | 0.73724 | 0.17211 | 0.07366 | 0.26450 |  |
| MeOH sp. | 0.00143 | 0.99936 | 0.97583 | 0.03112 | 0.01005 | 0.05720 | 0.99847 |

Table S10. Results from the Kruskal Wallis test with Nemenyi post-hoc testing of nucelosides and similar metabolites in the aqueous extracts, analyzed in positive ionization, with the exception of uridine that was detected in negative ionization

| Herbipoline, p= 1.204e-9 | | | | | | | |
| --- | --- | --- | --- | --- | --- | --- | --- |
|  | EtOH extr.1 | EtOH extr.2 | EtOH sp. | Frozen | Flash frozen | MeOH extr.1 | MeOH extr.2 |
| EtOH extr.2 | 0.00127 |  |  |  |  |  |  |
| EtOH sp. | 0.04937 | 0.97082 |  |  |  |  |  |
| Frozen | 0.99994 | 0.00024 | 0.01459 |  |  |  |  |
| Flash frozen | 0.99977 | 0.00805 | 0.16992 | 0.98972 |  |  |  |
| MeOH extr.1 | 0.99936 | 0.01067 | 0.20243 | 0.98272 | 1.00000 |  |  |
| MeOH extr.2 | 0.00184 | 1.00000 | 0.98272 | 0.00036 | 0.01110 | 0.01459 |  |
| MeOH sp. | 0.00105 | 1.00000 | 0.96301 | 0.00019 | 0.00682 | 0.00909 | 1.00000 |
| Methylguanine, p= 0.03723 | | | | | | | |
|  | EtOH extr.1 | EtOH extr.2 | EtOH sp. | Frozen | Flash frozen | MeOH extr.1 | MeOH extr.2 |
| EtOH extr.2 | 0.34707 |  |  |  |  |  |  |
| EtOH sp. | 0.82082 | 0.99605 |  |  |  |  |  |
| Frozen | 1.00000 | 0.47089 | 0.90509 |  |  |  |  |
| Flash frozen | 1.00000 | 0.37483 | 0.84339 | 1.00000 |  |  |  |
| MeOH extr.1 | 0.99986 | 0.64182 | 0.96900 | 1.00000 | 0.99993 |  |  |
| MeOH extr.2 | 0.41084 | 1.00000 | 0.99847 | 0.54072 | 0.44054 | 0.70888 |  |
| MeOH sp. | 0.28244 | 1.00000 | 0.99050 | 0.39627 | 0.30743 | 0.56420 | 1.00000 |
| Guanine, p= 1.413e-7 | | | | | | | |
|  | EtOH extr.1 | EtOH extr.2 | EtOH sp. | Frozen | Flash frozen | MeOH extr.1 | MeOH extr.2 |
| EtOH extr.2 | 0.00006 |  |  |  |  |  |  |
| EtOH sp. | 0.96900 | 0.00566 |  |  |  |  |  |
| Frozen | 0.99999 | 0.00024 | 0.99523 |  |  |  |  |
| Flash frozen | 0.99973 | 0.00060 | 0.99936 | 1.00000 |  |  |  |
| MeOH extr.1 | 0.54072 | 0.09383 | 0.98799 | 0.73025 | 0.84339 |  |  |
| MeOH extr.2 | 0.00108 | 0.99863 | 0.04543 | 0.00338 | 0.00726 | 0.36083 |  |
| MeOH sp. | 0.99050 | 0.00271 | 1.00000 | 0.99926 | 0.99996 | 0.96301 | 0.02511 |
| Adenosine, p= 7.470e-9 | | | | | | | |
|  | EtOH extr.1 | EtOH extr.2 | EtOH sp. | Frozen | Flash frozen | MeOH extr.1 | MeOH extr.2 |
| EtOH extr.2 | 0.00520 |  |  |  |  |  |  |
| EtOH sp. | 0.99893 | 0.03968 |  |  |  |  |  |
| Frozen | 0.98148 | 0.00009 | 0.78406 |  |  |  |  |
| Flash frozen | 0.98972 | 0.08326 | 1.00000 | 0.61875 |  |  |  |
| MeOH extr.1 | 0.18107 | 0.94013 | 0.52509 | 0.01178 | 0.70163 |  |  |
| MeOH extr.2 | 0.00009 | 0.98272 | 0.00136 | 6.47E-07 | 0.00385 | 0.41084 |  |
| MeOH sp. | 0.22018 | 0.91295 | 0.58766 | 0.01606 | 0.75778 | 1.00000 | 0.35392 |
| Deoxyadenosine, p= 5.362e-9 | | | | | | | |
|  | EtOH extr.1 | EtOH extr.2 | EtOH sp. | Frozen | Flash frozen | MeOH extr.1 | MeOH extr.2 |
| EtOH extr.2 | 0.09109 |  |  |  |  |  |  |
| EtOH sp. | 0.95624 | 0.00226 |  |  |  |  |  |
| Frozen | 0.45565 | 0.00003 | 0.98272 |  |  |  |  |
| Flash frozen | 0.96709 | 0.00283 | 1.00000 | 0.97583 |  |  |  |
| MeOH extr.1 | 0.85924 | 0.84878 | 0.19512 | 0.01377 | 0.22018 |  |  |
| MeOH extr.2 | 0.02090 | 0.99968 | 0.00027 | 2.45E-06 | 0.00035 | 0.54072 |  |
| MeOH sp. | 0.95126 | 0.69432 | 0.33358 | 0.03338 | 0.36780 | 0.99999 | 0.36083 |
| 5´-methylthioadenosine, p= 5.418e-9 | | | | | | | |
|  | EtOH extr.1 | EtOH extr.2 | EtOH sp. | Frozen | Flash frozen | MeOH extr.1 | MeOH extr.2 |
| EtOH extr.2 | 8.76E-06 |  |  |  |  |  |  |
| EtOH sp. | 0.07366 | 0.34029 |  |  |  |  |  |
| Frozen | 0.00035 | 0.99523 | 0.82661 |  |  |  |  |
| Flash frozen | 0.05019 | 0.42560 | 1.00000 | 0.88806 |  |  |  |
| MeOH extr.1 | 0.99847 | 0.00020 | 0.31386 | 0.00477 | 0.24170 |  |  |
| MeOH extr.2 | 1.11E-05 | 1.00000 | 0.36780 | 0.99675 | 0.45565 | 0.00024 |  |
| MeOH sp. | 0.47089 | 0.04106 | 0.98799 | 0.27040 | 0.97257 | 0.86431 | 0.04697 |
| Methylcytosine, p= 8.777e-10 | | | | | | | |
|  | EtOH extr.1 | EtOH extr.2 | EtOH sp. | Frozen | Flash frozen | MeOH extr.1 | MeOH extr.2 |
| EtOH extr.2 | 0.05813 |  |  |  |  |  |  |
| EtOH sp. | 0.01351 | 0.99980 |  |  |  |  |  |
| Frozen | 0.97082 | 0.00161 | 0.00021 |  |  |  |  |
| Flash frozen | 0.99971 | 0.01225 | 0.00216 | 0.99948 |  |  |  |
| MeOH extr.1 | 0.99997 | 0.14326 | 0.04106 | 0.87172 | 0.99124 |  |  |
| MeOH extr.2 | 0.03057 | 1.00000 | 1.00000 | 0.00065 | 0.00566 | 0.08326 |  |
| MeOH sp. | 0.00345 | 0.99124 | 0.99995 | 0.00004 | 0.00045 | 0.01225 | 0.99863 |
| Methylcytidine, p= 1.57e-9 | | | | | | | |
|  | EtOH extr.1 | EtOH extr.2 | EtOH sp. | Frozen | Flash frozen | MeOH extr.1 | MeOH extr.2 |
| EtOH extr.2 | 0.01940 |  |  |  |  |  |  |
| EtOH sp. | 0.00420 | 0.99988 |  |  |  |  |  |
| Frozen | 0.99992 | 0.06708 | 0.01800 |  |  |  |  |
| Flash frozen | 1.00000 | 0.02090 | 0.00457 | 0.99994 |  |  |  |
| MeOH extr.1 | 0.99993 | 0.06500 | 0.01733 | 1.00000 | 0.99995 |  |  |
| MeOH extr.2 | 0.00668 | 0.99999 | 1.00000 | 0.02699 | 0.00726 | 0.02603 |  |
| MeOH sp. | 0.00011 | 0.90509 | 0.98972 | 0.00067 | 0.00012 | 0.00063 | 0.97735 |
| Uridine, p= 4.467e-7 | | | | | | | |
|  | EtOH extr.1 | EtOH extr.2 | EtOH sp. | Frozen | Flash frozen | MeOH extr.1 | MeOH extr.2 |
| EtOH extr.2 | 0.00004 |  |  |  |  |  |  |
| EtOH sp. | 1.00000 | 0.00015 |  |  |  |  |  |
| Frozen | 0.98972 | 0.00207 | 0.99893 |  |  |  |  |
| Flash frozen | 0.99318 | 0.00164 | 0.99944 | 1.00000 |  |  |  |
| MeOH extr.1 | 0.99761 | 0.00098 | 0.99990 | 1.00000 | 1.00000 |  |  |
| MeOH extr.2 | 0.00641 | 0.94589 | 0.01606 | 0.09664 | 0.08326 | 0.05907 |  |
| MeOH sp. | 0.37483 | 0.14326 | 0.54855 | 0.89679 | 0.87413 | 0.81493 | 0.81493 |

Table S11. Results from the Kruskal Wallis test with Nemenyi post-hoc testing of the quaternary amines and similar metabolites in the aqueous extracts, analyzed in positive ionization.

| L-Carnitine, p= 1.905e-10 | | | | | | | |
| --- | --- | --- | --- | --- | --- | --- | --- |
|  | EtOH extr.1 | EtOH extr.2 | EtOH sp. | Frozen | Flash frozen | MeOH extr.1 | MeOH extr.2 |
| EtOH extr.2 | 0.02603 |  |  |  |  |  |  |
| EtOH sp. | 0.11489 | 0.99958 |  |  |  |  |  |
| Frozen | 1.00000 | 0.02250 | 0.10245 |  |  |  |  |
| Flash frozen | 0.99911 | 0.00330 | 0.02129 | 0.99948 |  |  |  |
| MeOH extr.1 | 0.99999 | 0.06198 | 0.22280 | 0.99998 | 0.98888 |  |  |
| MeOH extr.2 | 0.01800 | 1.00000 | 0.99847 | 0.01546 | 0.00211 | 0.04468 |  |
| MeOH sp. | 0.00002 | 0.69432 | 0.34707 | 0.00002 | 9.82E-07 | 0.00009 | 0.76447 |
| Acetylcarnitine, p= 5.538e-10 | | | | | | | |
|  | EtOH extr.1 | EtOH extr.2 | EtOH sp. | Frozen | Flash frozen | MeOH extr.1 | MeOH extr.2 |
| EtOH extr.2 | 0.04247 |  |  |  |  |  |  |
| EtOH sp. | 0.01178 | 0.99993 |  |  |  |  |  |
| Frozen | 0.99998 | 0.10853 | 0.03579 |  |  |  |  |
| Flash frozen | 1.00000 | 0.03112 | 0.00821 | 0.99986 |  |  |  |
| MeOH extr.1 | 0.99968 | 0.15927 | 0.05720 | 1.00000 | 0.99893 |  |  |
| MeOH extr.2 | 0.00965 | 0.99983 | 1.00000 | 0.03004 | 0.00668 | 0.04856 |  |
| MeOH sp. | 1.11E-05 | 0.49399 | 0.75778 | 0.00006 | 6.50E-06 | 0.00013 | 0.79041 |
| Dehydroxycarnitine, p= 2.015e-10 | | | | | | | |
|  | EtOH extr.1 | EtOH extr.2 | EtOH sp. | Frozen | Flash frozen | MeOH extr.1 | MeOH extr.2 |
| EtOH extr.2 | 0.01733 |  |  |  |  |  |  |
| EtOH sp. | 0.19036 | 0.98972 |  |  |  |  |  |
| Frozen | 1.00000 | 0.00838 | 0.11653 |  |  |  |  |
| Flash frozen | 0.99011 | 0.00065 | 0.01766 | 0.99828 |  |  |  |
| MeOH extr.1 | 1.00000 | 0.03579 | 0.30107 | 0.99987 | 0.96194 |  |  |
| MeOH extr.2 | 0.02797 | 1.00000 | 0.99675 | 0.01404 | 0.00121 | 0.05537 |  |
| MeOH sp. | 0.00008 | 0.90100 | 0.38192 | 0.00003 | 9.82E-07 | 0.00024 | 0.83790 |
| Betaine, p= 1.920e-10 | | | | | | | |
|  | EtOH extr.1 | EtOH extr.2 | EtOH sp. | Frozen | Flash frozen | MeOH extr.1 | MeOH extr.2 |
| EtOH extr.2 | 0.03397 |  |  |  |  |  |  |
| EtOH sp. | 0.02557 | 1.00000 |  |  |  |  |  |
| Frozen | 1.00000 | 0.03968 | 0.03004 |  |  |  |  |
| Flash frozen | 0.99749 | 0.00309 | 0.00216 | 0.99605 |  |  |  |
| MeOH extr.1 | 0.99992 | 0.10698 | 0.08452 | 0.99997 | 0.95973 |  |  |
| MeOH extr.2 | 0.05273 | 1.00000 | 1.00000 | 0.06099 | 0.00542 | 0.15312 |  |
| MeOH sp. | 0.00002 | 0.62646 | 0.68696 | 0.00003 | 4.99E-07 | 0.00015 | 0.52509 |
| Prolinebetaine, p= 2.368e-10 | | | | | | | |
|  | EtOH extr.1 | EtOH extr.2 | EtOH sp. | Frozen | Flash frozen | MeOH extr.1 | MeOH extr.2 |
| EtOH extr.2 | 0.08452 |  |  |  |  |  |  |
| EtOH sp. | 0.00946 | 0.99706 |  |  |  |  |  |
| Frozen | 1.00000 | 0.05537 | 0.00542 |  |  |  |  |
| Flash frozen | 0.99973 | 0.01977 | 0.00146 | 0.99998 |  |  |  |
| MeOH extr.1 | 1.00000 | 0.13572 | 0.01800 | 0.99998 | 0.99774 |  |  |
| MeOH extr.2 | 0.07253 | 1.00000 | 0.99828 | 0.04697 | 0.01637 | 0.11818 |  |
| MeOH sp. | 0.00003 | 0.47089 | 0.89248 | 1.49E-05 | 2.52E-06 | 0.00008 | 0.50950 |
| Alaninebetaine, p= 8.206e-10 | | | | | | | |
|  | EtOH extr.1 | EtOH extr.2 | EtOH sp. | Frozen | Flash frozen | MeOH extr.1 | MeOH extr.2 |
| EtOH extr.2 | 0.00641 |  |  |  |  |  |  |
| EtOH sp. | 0.31386 | 0.86431 |  |  |  |  |  |
| Frozen | 0.99973 | 0.00093 | 0.10853 |  |  |  |  |
| Flash frozen | 0.99523 | 0.00028 | 0.05187 | 0.99999 |  |  |  |
| MeOH extr.1 | 1.00000 | 0.00726 | 0.33358 | 0.99958 | 0.99375 |  |  |
| MeOH extr.2 | 0.04393 | 0.99916 | 0.99258 | 0.00891 | 0.00323 | 0.04856 |  |
| MeOH sp. | 0.00085 | 0.99963 | 0.55637 | 0.00009 | 0.00002 | 0.00098 | 0.96085 |
| Choline, p= 8.160e-11 | | | | | | | |
|  | EtOH extr.1 | EtOH extr.2 | EtOH sp. | Frozen | Flash frozen | MeOH extr.1 | MeOH extr.2 |
| EtOH extr.2 | 0.04393 |  |  |  |  |  |  |
| EtOH sp. | 0.00005 | 0.70163 |  |  |  |  |  |
| Frozen | 0.95126 | 0.51729 | 0.00697 |  |  |  |  |
| Flash frozen | 0.31386 | 0.99258 | 0.19997 | 0.94863 |  |  |  |
| MeOH extr.1 | 0.96900 | 0.45565 | 0.00498 | 1.00000 | 0.92394 |  |  |
| MeOH extr.2 | 0.00010 | 0.77761 | 1.00000 | 0.01088 | 0.25868 | 0.00788 |  |
| MeOH sp. | 1.23E-07 | 0.09664 | 0.95380 | 0.00006 | 0.00757 | 0.00004 | 0.92039 |
| Choline Sulfate, p= 3.088e-10 | | | | | | | |
|  | EtOH extr.1 | EtOH extr.2 | EtOH sp. | Frozen | Flash frozen | MeOH extr.1 | MeOH extr.2 |
| EtOH extr.2 | 0.00821 |  |  |  |  |  |  |
| EtOH sp. | 0.06708 | 0.99808 |  |  |  |  |  |
| Frozen | 1.00000 | 0.00788 | 0.06500 |  |  |  |  |
| Flash frozen | 0.99966 | 0.00116 | 0.01404 | 0.99971 |  |  |  |
| MeOH extr.1 | 1.00000 | 0.01110 | 0.08452 | 1.00000 | 0.99905 |  |  |
| MeOH extr.2 | 0.07834 | 0.99675 | 1.00000 | 0.07597 | 0.01701 | 0.09806 |  |
| MeOH sp. | 0.00005 | 0.93073 | 0.57985 | 0.00004 | 3.44E-06 | 0.00007 | 0.54072 |
| Arsenobetaine, p= 1.772e-10 | | | | | | | |
|  | EtOH extr.1 | EtOH extr.2 | EtOH sp. | Frozen | Flash frozen | MeOH extr.1 | MeOH extr.2 |
| EtOH extr.2 | 0.04856 |  |  |  |  |  |  |
| EtOH sp. | 0.00296 | 0.99258 |  |  |  |  |  |
| Frozen | 0.99992 | 0.14326 | 0.01325 |  |  |  |  |
| Flash frozen | 1.00000 | 0.05628 | 0.00360 | 0.99997 |  |  |  |
| MeOH extr.1 | 0.99586 | 0.29167 | 0.03901 | 0.99998 | 0.99735 |  |  |
| MeOH extr.2 | 0.00821 | 0.99944 | 0.99999 | 0.03223 | 0.00985 | 0.08452 |  |
| MeOH sp. | 2.95E-06 | 0.30743 | 0.82661 | 0.00002 | 3.88E-06 | 0.00013 | 0.66458 |

Table S12. Results from the Kruskal Wallis test with Nemenyi post-hoc testing of lipids and in the aqueous extracts, analyzed in negative ionization.

| LPCh(16:1), p= 5.853e-9 | | | | | | | |
| --- | --- | --- | --- | --- | --- | --- | --- |
|  | EtOH extr.1 | EtOH extr.2 | EtOH sp. | MeOH extr.1 | MeOH extr.2 | MeOH sp. | Frozen |
| EtOH extr.2 | 0.08580 |  |  |  |  |  |  |
| EtOH sp. | 1.68E-06 | 0.16347 |  |  |  |  |  |
| MeOH extr.1 | 1.00000 | 0.11168 | 2.95E-06 |  |  |  |  |
| MeOH extr.2 | 0.17655 | 0.99999 | 0.07834 | 0.22018 |  |  |  |
| MeOH sp. | 0.00005 | 0.53290 | 0.99808 | 0.00008 | 0.34029 |  |  |
| Frozen | 0.17211 | 0.99999 | 0.08077 | 0.21500 | 1.00000 | 0.34707 |  |
| Flash frozen | 0.47857 | 0.99124 | 0.01488 | 0.54855 | 0.99944 | 0.10545 | 0.99936 |
| LPCh(18:1), p= 1.441e-7 | | | | | | | |
|  | EtOH extr.1 | EtOH extr.2 | EtOH sp. | MeOH extr.1 | MeOH extr.2 | MeOH sp. | Frozen |
| EtOH extr.2 | 0.11818 |  |  |  |  |  |  |
| EtOH sp. | 0.00030 | 0.70163 |  |  |  |  |  |
| MeOH extr.1 | 1.00000 | 0.11168 | 0.00027 |  |  |  |  |
| MeOH extr.2 | 0.18107 | 1.00000 | 0.57985 | 0.17211 |  |  |  |
| MeOH sp. | 0.00216 | 0.92739 | 0.99980 | 0.00197 | 0.85924 |  |  |
| Frozen | 0.97424 | 0.67955 | 0.01606 | 0.97082 | 0.79041 | 0.06708 |  |
| Flash frozen | 1.00000 | 0.18107 | 0.00067 | 1.00000 | 2.65E-01 | 0.00438 | 0.99193 |
| LPE(16:1), p= 5.286e-9 | | | | | | | |
|  | EtOH extr.1 | EtOH extr.2 | EtOH sp. | MeOH extr.1 | MeOH extr.2 | MeOH sp. | Frozen |
| EtOH extr.2 | 0.00216 |  |  |  |  |  |  |
| EtOH sp. | 9.40E-08 | 0.49399 |  |  |  |  |  |
| MeOH extr.1 | 0.99936 | 0.01669 | 2.30E-06 |  |  |  |  |
| MeOH extr.2 | 0.00788 | 0.99998 | 0.28244 | 0.04856 |  |  |  |
| MeOH sp. | 0.00368 | 1.00000 | 0.40353 | 0.02603 | 1.00000 |  |  |
| Frozen | 0.07834 | 0.96509 | 0.04856 | 0.28244 | 0.99642 | 0.98389 |  |
| Flash frozen | 0.47857 | 0.53290 | 0.00237 | 0.83231 | 7.58E-01 | 0.62646 | 0.98888 |
| LPE(17:0), p= 1.028e-8 | | | | | | | |
|  | EtOH extr.1 | EtOH extr.2 | EtOH sp. | MeOH extr.1 | MeOH extr.2 | MeOH sp. | Frozen |
| EtOH extr.2 | 0.00017 |  |  |  |  |  |  |
| EtOH sp. | 1.91E-06 | 0.98799 |  |  |  |  |  |
| MeOH extr.1 | 0.99706 | 0.00338 | 0.00007 |  |  |  |  |
| MeOH extr.2 | 0.00098 | 0.99993 | 0.90908 | 0.01432 |  |  |  |
| MeOH sp. | 0.00821 | 0.98272 | 0.61100 | 0.07597 | 0.99944 |  |  |
| Frozen | 0.22544 | 0.42560 | 0.06099 | 0.66458 | 0.69432 | 0.94589 |  |
| Flash frozen | 6.03E-01 | 0.12497 | 0.00855 | 0.95126 | 0.29478 | 0.64944 | 0.99879 |
| LPG(16:0), p= 4.703e-11 | | | | | | | |
|  | EtOH extr.1 | EtOH extr.2 | EtOH sp. | MeOH extr.1 | MeOH extr.2 | MeOH sp. | Frozen |
| EtOH extr.2 | 0.00757 |  |  |  |  |  |  |
| EtOH sp. | 4.52E-06 | 0.73025 |  |  |  |  |  |
| MeOH extr.1 | 0.99997 | 0.02511 | 0.00003 |  |  |  |  |
| MeOH extr.2 | 0.01606 | 1.00000 | 0.58766 | 0.04856 |  |  |  |
| MeOH sp. | 4.67E-07 | 0.45565 | 0.99992 | 0.00000 | 0.32036 |  |  |
| Frozen | 0.22544 | 0.94013 | 0.09383 | 0.42560 | 0.98017 | 0.02899 |  |
| Flash frozen | 9.05E-01 | 0.28244 | 0.00197 | 0.98272 | 0.41084 | 0.00035 | 0.94589 |
| LPG(17:0), p= 3.613e-9 | | | | | | | |
|  | EtOH extr.1 | EtOH extr.2 | EtOH sp. | MeOH extr.1 | MeOH extr.2 | MeOH sp. | Frozen |
| EtOH extr.2 | 0.00641 |  |  |  |  |  |  |
| EtOH sp. | 1.05E-05 | 0.84339 |  |  |  |  |  |
| MeOH extr.1 | 0.99999 | 0.01800 | 0.00005 |  |  |  |  |
| MeOH extr.2 | 0.02797 | 0.99988 | 0.57985 | 0.06708 |  |  |  |
| MeOH sp. | 9.86E-06 | 0.83790 | 1.00000 | 0.00004 | 0.57203 |  |  |
| Frozen | 0.41084 | 0.78406 | 0.05907 | 0.61100 | 0.95380 | 0.05720 |  |
| Flash frozen | 5.10E-01 | 0.69432 | 0.03835 | 0.70888 | 0.91295 | 0.03705 | 1.00000 |
| PC(32:1), p= 4.055e-11 | | | | | | | |
|  | EtOH extr.1 | EtOH extr.2 | EtOH sp. | MeOH extr.1 | MeOH extr.2 | MeOH sp. | Frozen |
| EtOH extr.2 | 0.95859 |  |  |  |  |  |  |
| EtOH sp. | 0.00007 | 0.00726 |  |  |  |  |  |
| MeOH extr.1 | 0.98705 | 0.49399 | 0.00000 |  |  |  |  |
| MeOH extr.2 | 0.97424 | 1.00000 | 0.00520 | 0.55637 |  |  |  |
| MeOH sp. | 0.00005 | 0.00590 | 1.00000 | 4.38E-07 | 0.00420 |  |  |
| Frozen | 0.01606 | 0.29478 | 0.89248 | 0.00050 | 0.24728 | 0.86928 |  |
| Flash frozen | 0.13945 | 0.77761 | 0.44054 | 0.00965 | 0.72319 | 0.40353 | 0.99566 |
| PC(36:2), p= 2.672e-11 | | | | | | | |
|  | EtOH extr.1 | EtOH extr.2 | EtOH sp. | MeOH extr.1 | MeOH extr.2 | MeOH sp. | Frozen |
| EtOH extr.2 | 0.98272 |  |  |  |  |  |  |
| EtOH sp. | 0.00007 | 0.00402 |  |  |  |  |  |
| MeOH extr.1 | 0.98389 | 0.57985 | 4.99E-07 |  |  |  |  |
| MeOH extr.2 | 0.99893 | 0.99998 | 0.00103 | 0.79668 |  |  |  |
| MeOH sp. | 0.00007 | 0.00420 | 1.00000 | 5.32E-07 | 0.00108 |  |  |
| Frozen | 0.05907 | 0.45565 | 0.66458 | 0.00247 | 0.25294 | 0.67209 |  |
| Flash frozen | 0.08842 | 0.55637 | 0.56420 | 0.00438 | 3.34E-01 | 0.57203 | 1.00000 |
| PE (33:1), p= 5.416e-10 | | | | | | | |
|  | EtOH extr.1 | EtOH extr.2 | EtOH sp. | MeOH extr.1 | MeOH extr.2 | MeOH sp. | Frozen |
| EtOH extr.2 | 0.99999 |  |  |  |  |  |  |
| EtOH sp. | 0.00108 | 0.00323 |  |  |  |  |  |
| MeOH extr.1 | 0.95380 | 0.86431 | 5.11E-06 |  |  |  |  |
| MeOH extr.2 | 0.99477 | 0.96900 | 0.00003 | 0.99997 |  |  |  |
| MeOH sp. | 0.01733 | 0.04106 | 0.99605 | 0.00020 | 0.00093 |  |  |
| Frozen | 0.07141 | 0.14326 | 0.93397 | 0.00150 | 0.00590 | 0.99980 |  |
| Flash frozen | 0.25868 | 0.41819 | 0.66458 | 0.01178 | 3.71E-02 | 0.97424 | 0.99944 |
| PMeE (34:2), p= 3.316e-10 | | | | | | | |
|  | EtOH extr.1 | EtOH extr.2 | EtOH sp. | MeOH extr.1 | MeOH extr.2 | MeOH sp. | Frozen |
| EtOH extr.2 | 0.99706 |  |  |  |  |  |  |
| EtOH sp. | 0.00172 | 0.02250 |  |  |  |  |  |
| MeOH extr.1 | 0.95624 | 0.61875 | 9.86E-06 |  |  |  |  |
| MeOH extr.2 | 0.99990 | 0.95380 | 0.00028 | 0.99735 |  |  |  |
| MeOH sp. | 0.00207 | 0.02603 | 1.00000 | 1.25E-05 | 0.00035 |  |  |
| Frozen | 0.06922 | 0.34029 | 0.96301 | 0.00150 | 0.01940 | 0.97082 |  |
| Flash frozen | 0.09109 | 0.40353 | 0.94013 | 0.00226 | 0.02699 | 0.95126 | 1.00000 |
| PG(32:2), p= 2.45e-9 | | | | | | | |
|  | EtOH extr.1 | EtOH extr.2 | EtOH sp. | MeOH extr.1 | MeOH extr.2 | MeOH sp. | Frozen |
| EtOH extr.2 | 0.57203 |  |  |  |  |  |  |
| EtOH sp. | 0.00021 | 0.15515 |  |  |  |  |  |
| MeOH extr.1 | 0.93073 | 0.04393 | 3.84E-07 |  |  |  |  |
| MeOH extr.2 | 0.97583 | 0.98799 | 0.01178 | 0.35392 |  |  |  |
| MeOH sp. | 0.00033 | 0.19512 | 1.00000 | 6.90E-07 | 0.01669 |  |  |
| Frozen | 0.61875 | 1.00000 | 0.13206 | 0.05360 | 0.99258 | 0.16775 |  |
| Flash frozen | 0.94013 | 0.99706 | 0.02169 | 0.25294 | 1.00000 | 0.03004 | 0.99847 |
| PG(34:1), p= 4.009e-10 | | | | | | | |
|  | EtOH extr.1 | EtOH extr.2 | EtOH sp. | MeOH extr.1 | MeOH extr.2 | MeOH sp. | Frozen |
| EtOH extr.2 | 0.98500 |  |  |  |  |  |  |
| EtOH sp. | 0.00037 | 0.01377 |  |  |  |  |  |
| MeOH extr.1 | 0.98799 | 0.62646 | 4.81E-06 |  |  |  |  |
| MeOH extr.2 | 1.00000 | 0.99523 | 0.00073 | 0.96900 |  |  |  |
| MeOH sp. | 0.00012 | 0.00566 | 1.00000 | 1.23E-06 | 0.00024 |  |  |
| Frozen | 0.16775 | 0.71607 | 0.63416 | 0.01325 | 0.23619 | 0.46325 |  |
| Flash frozen | 0.34029 | 0.89679 | 0.39627 | 0.04106 | 0.44054 | 0.25294 | 0.99997 |

Table S13. Results from the Kruskal Wallis test with Nemenyi post-hoc testing of lipids and in the organic extracts, analyzed in negative ionization.

| LPCh(17:0), p= 2.978e-7 | | | | | | | |
| --- | --- | --- | --- | --- | --- | --- | --- |
|  | EtOH extr.1 | EtOH extr.2 | EtOH sp. | Frozen | Flash frozen | MeOH extr.1 | MeOH extr.2 |
| EtOH extr.2 | 0.08580 |  |  |  |  |  |  |
| EtOH sp. | 1.68E-06 | 0.16347 |  |  |  |  |  |
| Frozen | 1.00000 | 0.11168 | 2.95E-06 |  |  |  |  |
| Flash frozen | 0.17655 | 0.99999 | 0.07834 | 0.22018 |  |  |  |
| MeOH extr.1 | 0.00005 | 0.53290 | 0.99808 | 0.00008 | 0.34029 |  |  |
| MeOH extr.2 | 0.17211 | 0.99999 | 0.08077 | 0.21500 | 1.00000 | 0.34707 |  |
| MeOH sp. | 0.47857 | 0.99124 | 0.01488 | 0.54855 | 0.99944 | 0.10545 | 0.99936 |
| LPCh(18:1), p=4.367e-7 | | | | | | | |
|  | EtOH extr.1 | EtOH extr.2 | EtOH sp. | Frozen | Flash frozen | MeOH extr.1 | MeOH extr.2 |
| EtOH extr.2 | 0.11818 |  |  |  |  |  |  |
| EtOH sp. | 0.00030 | 0.70163 |  |  |  |  |  |
| Frozen | 1.00000 | 0.11168 | 0.00027 |  |  |  |  |
| Flash frozen | 0.18107 | 1.00000 | 0.57985 | 0.17211 |  |  |  |
| MeOH extr.1 | 0.00216 | 0.92739 | 0.99980 | 0.00197 | 0.85924 |  |  |
| MeOH extr.2 | 0.97424 | 0.67955 | 0.01606 | 0.97082 | 0.79041 | 0.06708 |  |
| MeOH sp. | 1.00000 | 0.18107 | 0.00067 | 1.00000 | 2.65E-01 | 0.00438 | 0.99193 |
| LPE(16:1), p= 2.889e-9 | | | | | | | |
|  | EtOH extr.1 | EtOH extr.2 | EtOH sp. | Frozen | Flash frozen | MeOH extr.1 | MeOH extr.2 |
| EtOH extr.2 | 0.00216 |  |  |  |  |  |  |
| EtOH sp. | 9.40E-08 | 0.49399 |  |  |  |  |  |
| Frozen | 0.99936 | 0.01669 | 2.30E-06 |  |  |  |  |
| Flash frozen | 0.00788 | 0.99998 | 0.28244 | 0.04856 |  |  |  |
| MeOH extr.1 | 0.00368 | 1.00000 | 0.40353 | 0.02603 | 1.00000 |  |  |
| MeOH extr.2 | 0.07834 | 0.96509 | 0.04856 | 0.28244 | 0.99642 | 0.98389 |  |
| MeOH sp. | 0.47857 | 0.53290 | 0.00237 | 0.83231 | 7.58E-01 | 0.62646 | 0.98888 |
| LPMeE(16:1), p= 3.608e-9 | | | | | | | |
|  | EtOH extr.1 | EtOH extr.2 | EtOH sp. | Frozen | Flash frozen | MeOH extr.1 | MeOH extr.2 |
| EtOH extr.2 | 0.00017 |  |  |  |  |  |  |
| EtOH sp. | 1.91E-06 | 0.98799 |  |  |  |  |  |
| Frozen | 0.99706 | 0.00338 | 0.00007 |  |  |  |  |
| Flash frozen | 0.00098 | 0.99993 | 0.90908 | 0.01432 |  |  |  |
| MeOH extr.1 | 0.00821 | 0.98272 | 0.61100 | 0.07597 | 0.99944 |  |  |
| MeOH extr.2 | 0.22544 | 0.42560 | 0.06099 | 0.66458 | 0.69432 | 0.94589 |  |
| MeOH sp. | 6.03E-01 | 0.12497 | 0.00855 | 0.95126 | 0.29478 | 0.64944 | 0.99879 |
| LPG(15:0), p= 1.183e-7 | | | | | | | |
|  | EtOH extr.1 | EtOH extr.2 | EtOH sp. | Frozen | Flash frozen | MeOH extr.1 | MeOH extr.2 |
| EtOH extr.2 | 0.00757 |  |  |  |  |  |  |
| EtOH sp. | 4.52E-06 | 0.73025 |  |  |  |  |  |
| Frozen | 0.99997 | 0.02511 | 0.00003 |  |  |  |  |
| Flash frozen | 0.01606 | 1.00000 | 0.58766 | 0.04856 |  |  |  |
| MeOH extr.1 | 4.67E-07 | 0.45565 | 0.99992 | 0.00000 | 0.32036 |  |  |
| MeOH extr.2 | 0.22544 | 0.94013 | 0.09383 | 0.42560 | 0.98017 | 0.02899 |  |
| MeOH sp. | 9.05E-01 | 0.28244 | 0.00197 | 0.98272 | 0.41084 | 0.00035 | 0.94589 |
| LPG(16:0), p= 6.767e-8 | | | | | | | |
|  | EtOH extr.1 | EtOH extr.2 | EtOH sp. | Frozen | Flash frozen | MeOH extr.1 | MeOH extr.2 |
| EtOH extr.2 | 0.00641 |  |  |  |  |  |  |
| EtOH sp. | 1.05E-05 | 0.84339 |  |  |  |  |  |
| Frozen | 0.99999 | 0.01800 | 0.00005 |  |  |  |  |
| Flash frozen | 0.02797 | 0.99988 | 0.57985 | 0.06708 |  |  |  |
| MeOH extr.1 | 9.86E-06 | 0.83790 | 1.00000 | 0.00004 | 0.57203 |  |  |
| MeOH extr.2 | 0.41084 | 0.78406 | 0.05907 | 0.61100 | 0.95380 | 0.05720 |  |
| MeOH sp. | 5.10E-01 | 0.69432 | 0.03835 | 0.70888 | 0.91295 | 0.03705 | 1.00000 |
| PC(34:3), p=3.739e-7 | | | | | | | |
|  | EtOH extr.1 | EtOH extr.2 | EtOH sp. | Frozen | Flash frozen | MeOH extr.1 | MeOH extr.2 |
| EtOH extr.2 | 0.95859 |  |  |  |  |  |  |
| EtOH sp. | 0.00007 | 0.00726 |  |  |  |  |  |
| Frozen | 0.98705 | 0.49399 | 0.00000 |  |  |  |  |
| Flash frozen | 0.97424 | 1.00000 | 0.00520 | 0.55637 |  |  |  |
| MeOH extr.1 | 0.00005 | 0.00590 | 1.00000 | 4.38E-07 | 0.00420 |  |  |
| MeOH extr.2 | 0.01606 | 0.29478 | 0.89248 | 0.00050 | 0.24728 | 0.86928 |  |
| MeOH sp. | 0.13945 | 0.77761 | 0.44054 | 0.00965 | 0.72319 | 0.40353 | 0.99566 |
| PC(35:3), p= 5.131e-7 | | | | | | | |
|  | EtOH extr.1 | EtOH extr.2 | EtOH sp. | Frozen | Flash frozen | MeOH extr.1 | MeOH extr.2 |
| EtOH extr.2 | 0.98272 |  |  |  |  |  |  |
| EtOH sp. | 0.00007 | 0.00402 |  |  |  |  |  |
| Frozen | 0.98389 | 0.57985 | 4.99E-07 |  |  |  |  |
| Flash frozen | 0.99893 | 0.99998 | 0.00103 | 0.79668 |  |  |  |
| MeOH extr.1 | 0.00007 | 0.00420 | 1.00000 | 5.32E-07 | 0.00108 |  |  |
| MeOH extr.2 | 0.05907 | 0.45565 | 0.66458 | 0.00247 | 0.25294 | 0.67209 |  |
| MeOH sp. | 0.08842 | 0.55637 | 0.56420 | 0.00438 | 3.34E-01 | 0.57203 | 1.00000 |
| PE (36:5), p= 4.743e-6 | | | | | | | |
|  | EtOH extr.1 | EtOH extr.2 | EtOH sp. | Frozen | Flash frozen | MeOH extr.1 | MeOH extr.2 |
| EtOH extr.2 | 0.99999 |  |  |  |  |  |  |
| EtOH sp. | 0.00108 | 0.00323 |  |  |  |  |  |
| Frozen | 0.95380 | 0.86431 | 5.11E-06 |  |  |  |  |
| Flash frozen | 0.99477 | 0.96900 | 0.00003 | 0.99997 |  |  |  |
| MeOH extr.1 | 0.01733 | 0.04106 | 0.99605 | 0.00020 | 0.00093 |  |  |
| MeOH extr.2 | 0.07141 | 0.14326 | 0.93397 | 0.00150 | 0.00590 | 0.99980 |  |
| MeOH sp. | 0.25868 | 0.41819 | 0.66458 | 0.01178 | 3.71E-02 | 0.97424 | 0.99944 |
| PE-P (38:5), p= 2.534e-5 | | | | | | | |
|  | EtOH extr.1 | EtOH extr.2 | EtOH sp. | Frozen | Flash frozen | MeOH extr.1 | MeOH extr.2 |
| EtOH extr.2 | 0.99706 |  |  |  |  |  |  |
| EtOH sp. | 0.00172 | 0.02250 |  |  |  |  |  |
| Frozen | 0.95624 | 0.61875 | 9.86E-06 |  |  |  |  |
| Flash frozen | 0.99990 | 0.95380 | 0.00028 | 0.99735 |  |  |  |
| MeOH extr.1 | 0.00207 | 0.02603 | 1.00000 | 1.25E-05 | 0.00035 |  |  |
| MeOH extr.2 | 0.06922 | 0.34029 | 0.96301 | 0.00150 | 0.01940 | 0.97082 |  |
| MeOH sp. | 0.09109 | 0.40353 | 0.94013 | 0.00226 | 0.02699 | 0.95126 | 1.00000 |
| PG(34:1), p= 2.87e-5 | | | | | | | |
|  | EtOH extr.1 | EtOH extr.2 | EtOH sp. | Frozen | Flash frozen | MeOH extr.1 | MeOH extr.2 |
| EtOH extr.2 | 0.57203 |  |  |  |  |  |  |
| EtOH sp. | 0.00021 | 0.15515 |  |  |  |  |  |
| Frozen | 0.93073 | 0.04393 | 3.84E-07 |  |  |  |  |
| Flash frozen | 0.97583 | 0.98799 | 0.01178 | 0.35392 |  |  |  |
| MeOH extr.1 | 0.00033 | 0.19512 | 1.00000 | 6.90E-07 | 0.01669 |  |  |
| MeOH extr.2 | 0.61875 | 1.00000 | 0.13206 | 0.05360 | 0.99258 | 0.16775 |  |
| MeOH sp. | 0.94013 | 0.99706 | 0.02169 | 0.25294 | 1.00000 | 0.03004 | 0.99847 |
| PG(35:1), p= 2.695e-5 | | | | | | | |
|  | EtOH extr.1 | EtOH extr.2 | EtOH sp. | Frozen | Flash frozen | MeOH extr.1 | MeOH extr.2 |
| EtOH extr.2 | 0.98500 |  |  |  |  |  |  |
| EtOH sp. | 0.00037 | 0.01377 |  |  |  |  |  |
| Frozen | 0.98799 | 0.62646 | 4.81E-06 |  |  |  |  |
| Flash frozen | 1.00000 | 0.99523 | 0.00073 | 0.96900 |  |  |  |
| MeOH extr.1 | 0.00012 | 0.00566 | 1.00000 | 1.23E-06 | 0.00024 |  |  |
| MeOH extr.2 | 0.16775 | 0.71607 | 0.63416 | 0.01325 | 0.23619 | 0.46325 |  |
| MeOH sp. | 0.34029 | 0.89679 | 0.39627 | 0.04106 | 0.44054 | 0.25294 | 0.99997 |

Table S14. Results from the Kruskal Wallis test with Nemenyi post-hoc testing of peptides and in the organic extracts, analyzed in positive ionization.

| Barrettide A, p= 6.445e-8 | | | | | | | |
| --- | --- | --- | --- | --- | --- | --- | --- |
|  | EtOH extr.1 | EtOH extr.2 | EtOH sp. | Frozen | Flash frozen | MeOH extr.1 | MeOH extr.2 |
| EtOH extr.2 | 0.64182 |  |  |  |  |  |  |
| EtOH sp. | 0.16347 | 0.00037 |  |  |  |  |  |
| Frozen | 0.76447 | 0.01869 | 0.97424 |  |  |  |  |
| Flash frozen | 0.38192 | 0.00226 | 0.99988 | 0.99916 |  |  |  |
| MeOH extr.1 | 0.99193 | 0.98148 | 0.01546 | 0.24170 | 0.05907 |  |  |
| MeOH extr.2 | 0.78406 | 1.00000 | 0.00098 | 0.03835 | 0.00542 | 0.99642 |  |
| MeOH sp. | 0.20991 | 0.00060 | 1.00000 | 0.98705 | 0.99999 | 0.02250 | 0.00157 |
| Barrettide B, p= 7.461e-8 | | | | | | | |
|  | EtOH extr.1 | EtOH extr.2 | EtOH sp. | Frozen | Flash frozen | MeOH extr.1 | MeOH extr.2 |
| EtOH extr.2 | 0.62646 |  |  |  |  |  |  |
| EtOH sp. | 0.20991 | 0.00055 |  |  |  |  |  |
| Frozen | 0.76447 | 0.01733 | 0.98705 |  |  |  |  |
| Flash frozen | 0.46325 | 0.00338 | 0.99986 | 0.99983 |  |  |  |
| MeOH extr.1 | 0.98799 | 0.98500 | 0.01869 | 0.21500 | 0.07141 |  |  |
| MeOH extr.2 | 0.71607 | 1.00000 | 0.00098 | 0.02699 | 0.00566 | 0.99428 |  |
| MeOH sp. | 0.22018 | 0.00060 | 1.00000 | 0.98888 | 0.99990 | 0.02014 | 0.00108 |
| X3100, p= 6.084e-9 | | | | | | | |
|  | EtOH extr.1 | EtOH extr.2 | EtOH sp. | Frozen | Flash frozen | MeOH extr.1 | MeOH extr.2 |
| EtOH extr.2 | 0.85924 |  |  |  |  |  |  |
| EtOH sp. | 0.15515 | 0.00164 |  |  |  |  |  |
| Frozen | 0.11168 | 0.00093 | 1.00000 |  |  |  |  |
| Flash frozen | 0.12848 | 0.00119 | 1.00000 | 1.00000 |  |  |  |
| MeOH extr.1 | 0.99916 | 0.99193 | 0.03223 | 0.02090 | 0.02511 |  |  |
| MeOH extr.2 | 0.71607 | 1.00000 | 0.00055 | 0.00030 | 0.00039 | 0.96085 |  |
| MeOH sp. | 0.33358 | 0.00697 | 0.99995 | 0.99963 | 0.99983 | 0.09383 | 0.00259 |
| X3114, p= 4.793e-9 | | | | | | | |
|  | EtOH extr.1 | EtOH extr.2 | EtOH sp. | Frozen | Flash frozen | MeOH extr.1 | MeOH extr.2 |
| EtOH extr.2 | 0.54855 |  |  |  |  |  |  |
| EtOH sp. | 0.36083 | 0.00113 |  |  |  |  |  |
| Frozen | 0.25868 | 0.00052 | 1.00000 |  |  |  |  |
| Flash frozen | 0.23077 | 0.00041 | 1.00000 | 1.00000 |  |  |  |
| MeOH extr.1 | 0.98017 | 0.98148 | 0.03579 | 0.02014 | 0.01669 |  |  |
| MeOH extr.2 | 0.51729 | 1.00000 | 0.00093 | 0.00043 | 0.00033 | 0.97583 |  |
| MeOH sp. | 0.52509 | 0.00309 | 1.00000 | 0.99988 | 0.99973 | 0.07366 | 0.00259 |
| X3231, p= 1.498e-8 | | | | | | | |
|  | EtOH extr.1 | EtOH extr.2 | EtOH sp. | Frozen | Flash frozen | MeOH extr.1 | MeOH extr.2 |
| EtOH extr.2 | 0.35392 |  |  |  |  |  |  |
| EtOH sp. | 0.21500 | 0.00009 |  |  |  |  |  |
| Frozen | 0.76447 | 0.00402 | 0.98799 |  |  |  |  |
| Flash frozen | 0.49399 | 0.00077 | 0.99977 | 0.99992 |  |  |  |
| MeOH extr.1 | 0.99605 | 0.82661 | 0.03112 | 0.28857 | 0.11818 |  |  |
| MeOH extr.2 | 0.56420 | 0.99999 | 0.00039 | 0.01274 | 0.00283 | 0.94589 |  |
| MeOH sp. | 0.31386 | 0.00022 | 1.00000 | 0.99735 | 0.99999 | 0.05537 | 0.00089 |
| X3732, p= 1.185e-8 | | | | | | | |
|  | EtOH extr.1 | EtOH extr.2 | EtOH sp. | Frozen | Flash frozen | MeOH extr.1 | MeOH extr.2 |
| EtOH extr.2 | 0.00172 |  |  |  |  |  |  |
| EtOH sp. | 1.00000 | 0.00353 |  |  |  |  |  |
| Frozen | 0.97082 | 0.00001 | 0.93073 |  |  |  |  |
| Flash frozen | 0.98272 | 0.00002 | 0.95380 | 1.00000 |  |  |  |
| MeOH extr.1 | 0.63416 | 0.34707 | 0.75101 | 0.09383 | 0.11818 |  |  |
| MeOH extr.2 | 0.01132 | 0.99968 | 0.02090 | 0.00017 | 0.00026 | 0.67955 |  |
| MeOH sp. | 0.96900 | 0.06297 | 0.98972 | 0.44054 | 0.50174 | 0.99566 | 0.21500 |
